# Mechanistic perspective and functional activity of insulin in amylin aggregation†
†Electronic supplementary information (ESI) available. See DOI: 10.1039/c8sc00481a


**DOI:** 10.1039/c8sc00481a

**Published:** 2018-04-16

**Authors:** Michal Baram, Sharon Gilead, Ehud Gazit, Yifat Miller

**Affiliations:** a Department of Chemistry , Ben-Gurion University of the Negev , Be'er Sheva 84105 , Israel . Email: ymiller@bgu.ac.il; b The Ilse Katz Institute for Nanoscale Science & Technology , Ben-Gurion University of the Negev , Be'er Sheva 84105 , Israel; c Department of Molecular Microbiology and Biotechnology , Tel Aviv University , Tel Aviv 69978 , Israel . Email: ehudg@post.tau.ac.il; d Department of Materials Science and Engineering , Iby and Aladar Fleischman Faculty of Engineering , Tel Aviv University , Tel Aviv 69978 , Israel

## Abstract

Insulin is a key regulatory polypeptide that is secreted from pancreatic β-cells and has several important effects on the synthesis of lipids, regulation of enzymatic activities, blood glucose levels and the prevention of hyperglycemia. Insulin was demonstrated to self-assemble into ordered amyloid fibrils upon repeated injections, although the possible biological significance of the supramolecular structures is enigmatic. Amylin is also an amyloidogenic polypeptide that is secreted from pancreatic β-cells and plays an important role in glycemic regulation preventing post-prandial spikes in blood glucose levels. These two amyloidogenic proteins are secreted together from the pancreas and have the ability to interact and produce insulin–amylin aggregates. So far, the molecular architecture of insulin–amylin complexes at the atomic resolution has been unknown. The current work identifies for the first time the specific π–π interactions between Y16 in insulin and F19 in amylin that contribute to the stability of the insulin–amylin complex, by using experimental and molecular modeling techniques. We performed additional experiments that verify the functional activity of insulin in amylin aggregation. Our findings illustrate for the first time the specific interactions between insulin and amylin aggregates at the atomic resolution and provide a new mechanistic perspective on the effect of insulin on amylin aggregation and may pave the way towards pharmacological intervention in this process.

## Abstract 2

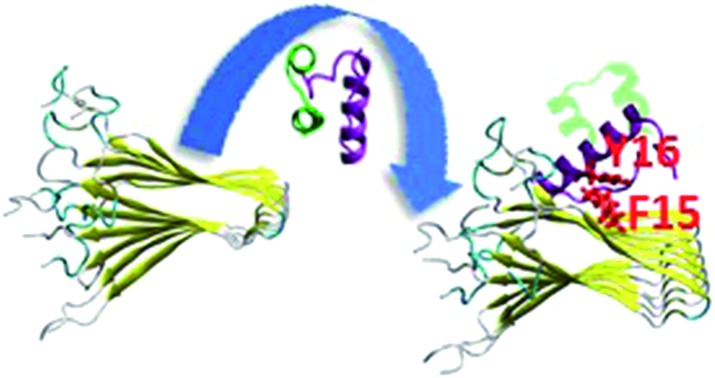
This work provides the first-ever complete atomic model of insulin–amylin aggregates, identifying the specific interactions that stabilize the insulin–amylin complex.

## Introduction

More than ninety years after its discovery^1^ and isolation, insulin is extensively used for the treatment of patients with type 1 diabetes and advanced-stage type 2 diabetes.^2^ It is well-established that insulin is secreted from pancreatic β-cells and acts as an agent that induces a decrease of glucose levels in the blood.^3^–^5^ The prevention of hypoglycemia by insulin-responsive glucagon has recently been reported.^6^ Amylin is a polypeptide that is also secreted by pancreatic β-cells together with insulin and is implicated in the development of type 2 diabetes (T2D) by forming amyloid aggregates that result in β-cell mass loss and consequently reduction of insulin secretion. The membranes play a role in amylin aggregation and cause toxicity to the islet pancreatic β-cells.^7^ Moreover, it has been shown that insulin suppresses the fiber-dependent membrane disruption by amylin, but not the pore-formation dependent membrane disruption.^8^ The effect of amylin on insulin secretion from β-cells has previously been studied,^9^ but the activation and the mechanisms underlying the effect of insulin on amylin aggregation remain controversial. Therefore, there is great interest in studying these interactions at the atomic resolution and understanding the functional activity of insulin in amylin aggregation.

Recently, there have been efforts to investigate the inhibition of amylin aggregation by organic and inorganic molecules.^10^–^12^ Insulin, which is a natural biological molecule that contains two chains, chain A and chain B, was shown to inhibit amylin aggregation,^13^–^16^ but only at short timescales.^17^ Furthermore, the regions within insulin and amylin polypeptides that interact with each other were identified.^11^ These studies proposed that the inhibition of amylin aggregation is due to the interactions between insulin and amylin. The interactions between insulin molecules and the amylin β-hairpin monomeric structure have been studied,^16^ but the interactions between insulin molecules and toxic amylin aggregates have not been investigated. While specific interaction domains have been proposed,^13^,^16^,^18^ a comprehensive study of the molecular mechanism and the functional activity of insulin in amylin aggregation has not been established. Moreover, we have previously shown that similar to other amyloids, amylin aggregates are also polymorphic,^19^ and therefore it is crucial to investigate the effect of insulin on various polymorphic amylin aggregates.

In this report, we address critical and unresolved questions concerning the probable mechanisms of the functional activity of insulin in polymorphic amylin aggregates: what are the specific binding sites between insulin and amylin toxic aggregates? Among all amylin polymorphic aggregates, which of them can interact with insulin? What is the dynamics of the effect of insulin on amylin aggregation? And what are the molecular mechanism pathways and functional activity of insulin in amylin aggregation? To resolve these issues, we have used a combination of experimental and computational approaches, where we introduce a comprehensive study of a mutation in the recognition domain hotspot between insulin and amylin aggregates.

## Results and discussion

### Insulin molecules prefer to interact uniquely with a specific amylin aggregate

Experimental studies showed that insulin inhibits amylin aggregation.^13^–^16^ The domains in which insulin binds to amylin and inhibits amylin aggregation have been suggested by two experimental studies.^13^,^18^ Our previous study suggested that the central domain of the insulin B chain (residues 9–20) binds to the first β-strand domain in amylin (residues 7–19). The second study proposed that the C-terminal of the insulin B chain (residues 22–29) binds to the second β-strand domain in amylin (residues 23–30), due to a recognition motif in amyloids. So far, these specific binding sites have not been investigated at the molecular level. Specifically, neither of these two binding sites has been investigated in polymorphic amylin aggregates. Therefore, this is the first study that examines at the molecular level the specific binding sites that facilitate the interaction of insulin and each of the four different amylin aggregates. The four different amylin aggregates have previously been studied by our group^19^ and are based on two experimental studies.^13^,^18^

**Fig. 1:**
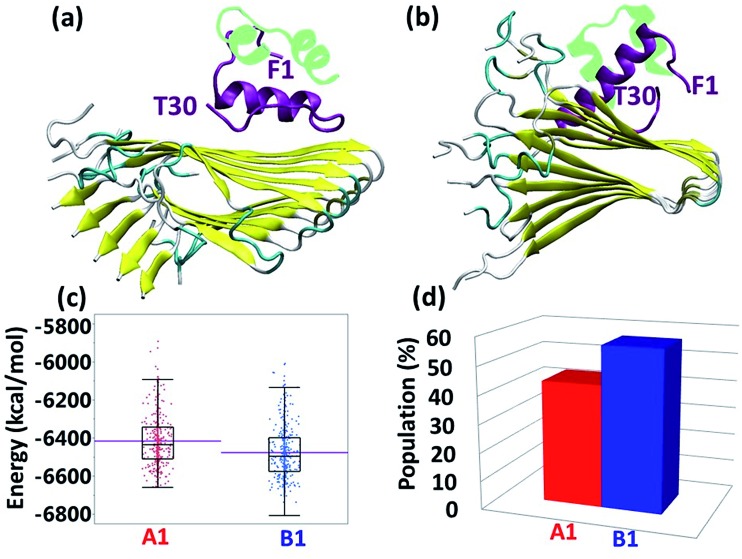
Simulated insulin–amylin aggregate models (a) A1 (b) B1. In models A1 and B1, the insulin molecule^48^ interacts with a unique amylin model, M1.^19^ (c) Scatter charts of the 500 conformations obtained from the GBMV energy values extracted from the last 5 ns of each model A1 and B1. The scatter charts represent the “histograms” of the number of conformations in the energy range. The averaged energy values are seen in the “boxes”. The *P* value *P*_A1_,_B1_ = 4.125 × 10^–12^. (d) Population analysis of models A1 and B1.

The constructed models that illustrate the two proposed binding sites between insulin and each one of the four amylin aggregates are detailed in ESI appendix, Fig. S1–S7.† Molecular dynamics (MD) simulations of the constructed models of insulin–amylin aggregates showed that the binding site in which the central domain of the insulin B chain (residues 9–20) binds to the first β-strand domain in amylin (residues 7–19) has been conserved during the MD simulations, although the insulin rotated along the twisted fibrillary structures. Fig. S8 and S9† illustrate that this binding domain has conserved partly or fully the specific interactions in the eight models. On the other hand, MD simulations showed that the binding domain in which the C-terminal of the insulin B chain (residues 22–29) binds to the second β-strand domain of amylin (residues 23–30) has not been conserved during the MD simulations (Fig. S10 and S11†). We therefore focused only on the eight models in which the binding site was conserved during the MD simulations. Conformational energy and population analyses indicate that among all these eight models, there are two models that are more stable and display mostly preferred conformations: models A1 and B1 (Fig. S12†). In these two conformational complexes, the insulin binds to a specific amylin aggregate – model M1. We thus conclude that among all four amylin aggregates, the insulin B chain (residues 9–20) prefers to interact with one specific amylin aggregate in a particular domain (residues 7–19), consistent with the previous findings in our peptide array experiment,^13^ in two different orientations: the first orientation is with residues 7–19, and the second with residues 19–7. These two models (models A1 and B1) are presented in Fig. 1a and b. Comparison between the conformational energies and populations of these two models shows that model B1 is slightly more preferred than model A1 (Fig. 1c and d). In summary, insulin molecules prefer to interact with a specific aggregate with a slightly preference for one orientation, as seen in model B1 (Fig. 1b).

### Identification of a specific aromatic interaction at the recognition interface of insulin–amylin aggregates

The specific recognition domain by which insulin binds amylin has previously been identified by our peptide-array analysis.^13^ The self-binding domain comprising residues 7–19 in the amylin polypeptide was detected at the recognition interface within the insulin B chain (residues 9–20). It was hypothesized, but not proven, that Y16 in the insulin B chain interacts *via* π-stacking with F15 in the amylin polypeptide. Since the most preferred insulin–amylin aggregate structural model is model B1, we chose to focus on this model.

**Fig. 2:**
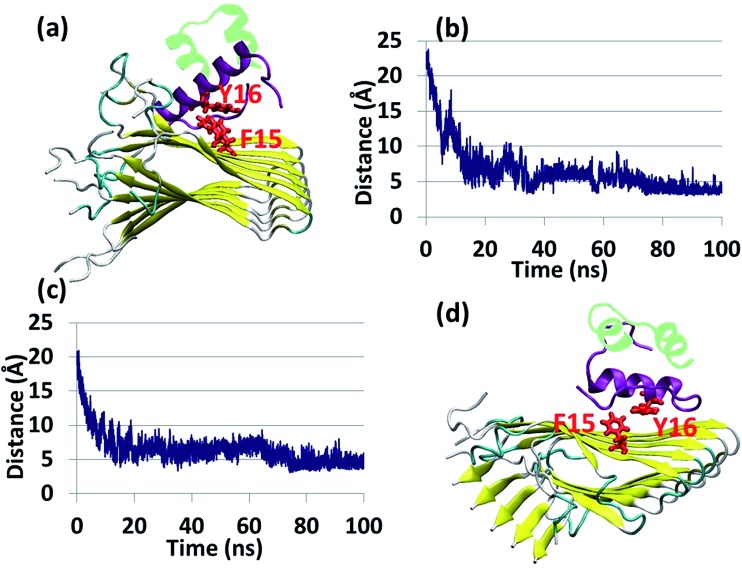
(a) Illustration of the π–π interactions between F15 of amylin and Y16 of insulin in model B1. (b) Distance between F15 in monomer 5 of the amylin aggregate and Y16 in IBC during the MD simulations in model B1. (c) Distance between F15 in monomer 6 of the amylin aggregate and Y16 in IBC during the MD simulations in model B1. (d) Illustration of the π–π interactions between F15 of amylin and Y16 of insulin in model A1.

Interestingly, the simulated model B1 of the insulin–amylin aggregate showed that this π-stacking interaction has been produced and conserved during the MD simulations (Fig. 2a–c). This π–π interaction was also illustrated in model A1 (Fig. 2d), but was not observed in any of the other insulin–amylin aggregate structural models that were examined in the current study. These findings indicate that the binding domain of residues 7–19 in the amylin aggregate has the ability to bind the insulin molecule in two different orientations of the B chain, and the specific interaction that stabilizes the contact between these two species in both orientations is the π-stacking between F15 of amylin and Y16 of insulin. Hydrophobic and electrostatic interactions were also found at the contact interface between the insulin B chain and amylin aggregates in model A1 (Fig. S13†), but these interactions make a minor contribution to the stability as compared to the π–π interactions.

It is known that aromatic interactions play an important role in the self-assembly of amyloid fibrils by producing interactions along the fibrillar axis of the amyloid^20^ and by producing interactions along fibrillar structures of other peptides as well.^21^,^22^ A recent computational study of the self-assembly of amylin showed that the distinctive residues that formed the initial contact between the amylin peptides are the aromatic residues F15 and F23.^23^ Therefore, these residues are the key domains in which potential inhibitors of amylin aggregation should be designed. Herein, we show that Y16 in the insulin B chain interacts with F15 of two adjacent amylin molecules in the aggregate, and therefore the Y16 may compete with the π stacking of two F15 residues (Fig. 2b and c).

### The specific aromatic interaction between F15 of amylin and Y16 of insulin plays a role in the inhibition of amylin aggregation

**Fig. 3:**
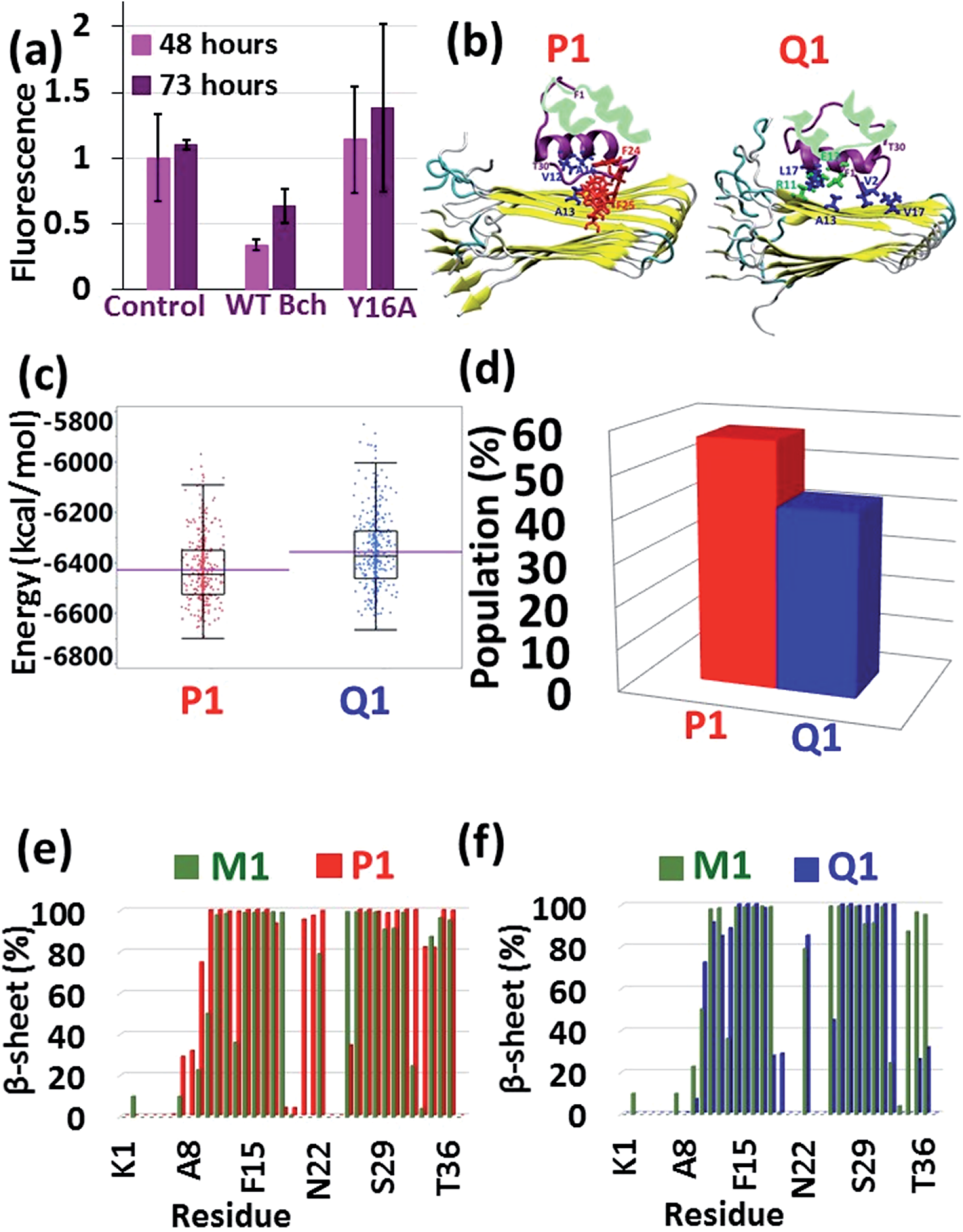
(a) ThT fluorescence measurements of amyloid formation by amylin alone (control), in the presence of a peptide derived from the amylin-binding region within the insulin B chain (wt Bch) and in the presence of a mutated form of Y16A. Error bars represent standard deviations of three independent repeats. (b) Simulated insulin–amylin aggregate models P1 and Q1, in which the Y16 is mutated to A16. Hydrophobic interactions (blue), electrostatic interactions (green) and π–π interactions (red) were produced during the MD simulations. (c) Scatter charts of the 500 conformations obtained from the GBMV energy values extracted from the last 5 ns of each model P1 and Q1. The scatter charts represent the “histograms” of the number of conformations in the energy range. The averaged energy values are seen in the “boxes”. The *P* value *P*_P1_,_Q1_ = 1.66 × 10^–12^. (d) Population analysis of models P1 and Q1. The percentage of β-strand properties of residues along the sequence of amylin in the amylin aggregate of (e) model M1 and in the insulin–amylin aggregate of model P1, and (f) model M1 and in the insulin–amylin aggregate of model Q1.

The results from the previous peptide-array analysis^13^ have driven us to further explore the recognition motif within the insulin B chain (residues ^10^HLVEALYLV^19^C) which binds amylin. We performed a systematic alanine scan of the recognition motif, where each of the amino acids was substituted with alanine. A series of peptides corresponding to this recognition motif with a single mutation into alanine along the sequence, excluding the alanine residue at position 14, were synthesized to produce a set of the wild-type (WT) peptide and nine mutated peptides (ESI appendix†). We tested the ability of each of these ten peptides to inhibit amylin fibrilization using the ThT binding assay (ESI appendix, Fig. S14†). Most of the substitutions were found to impair the inhibition ability of the WT peptide, but the mutation of Y16 to A16 illustrated the most dramatic effect with a typical distribution (Fig. 3a). These experimental results led us to suggest that the tyrosine residue at position 16 plays a central role in mediating the recognition and binding to amylin.

To prove these findings, we applied molecular modeling tools using the full-length insulin binding to the amylin aggregate. Since, the most populated insulin–amylin aggregate structural models are A1 and B1, we mutated Y16 to A16 in the insulin B chain in models A1 and B1 – producing models P1 and Q1, respectively. MD simulations for these two models were performed (Fig. 3b) and a comparison between these two models showed that model P1 is more stable and more populated than model Q1 (Fig. 3c and d). To determine the relative stability of model P1 over model Q1, a search for alternative interactions between F15 in amylin and other residues in the insulin B chain was performed in the two models. Interestingly, during the MD simulations a “bulky cluster” of aromatic residues produced several strong π–π interactions in model P1, therefore imparting stability to the insulin complex (Fig. 3b and S15†). This scenario of the “bulky cluster” of aromatic residues has not been found in model Q1. Moreover, the insulin molecules induce and stabilize the cross-β structure of the amylin aggregate in model P1, while in model Q1 the insulin molecules disrupted the cross-β structure in the C-termini of the amylin aggregates (Fig. 3e and f).

### The complexation of insulin molecules with amylin aggregates results in stabilization of the helix in the insulin B chain

**Fig. 4:**
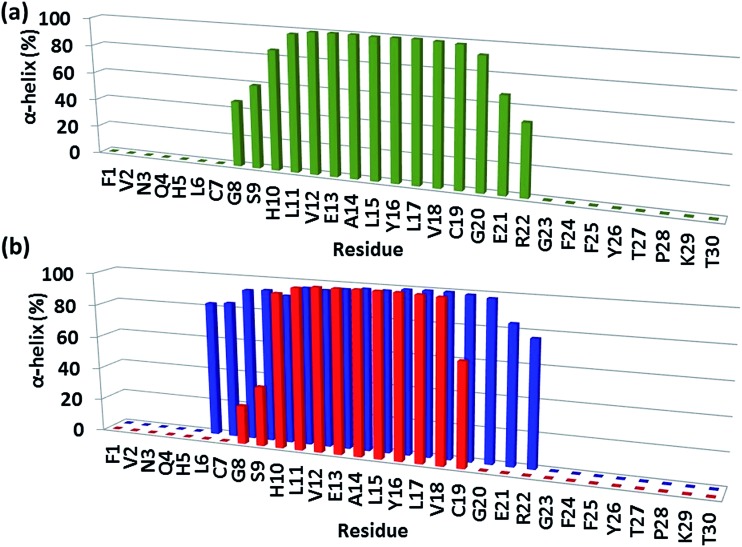
The percentage of α-helix properties of residues along the sequence of (a) insulin alone and (b) insulin in insulin–amylin aggregate model A1 (red) and in insulin–amylin aggregate model B1 (blue).

In model B1, the exclusive π–π interaction of F15 of the amylin aggregate and Y16 of the insulin B chain contributes to the stability of the complex and to the stability of the helical structure of chain B (Fig. 4). In model A1, besides this π stacking interaction, further hydrophobic and electrostatic interactions (Fig. S13†) appear at the contact interface between the amylin aggregate and insulin chain B. These hydrophobic and electrostatic interactions lead to the disruption of the helix of the insulin B chain in model A1 by decreasing the length of the ordered helical structure (Fig. 4). Interestingly, the mutation of Y16A in the insulin B chain for both models A1 and B1 also led to the formation of hydrophobic and electrostatic interactions that resulted in the disappearance of the helical structure in insulin B chain residues 20–22 (Fig. S15 and S16†).

The stability of helices within proteins has been extensively studied.^24^–^26^ However, to the best of our knowledge, so far studies on protein–protein interactions, in which the contact interface consists of a helical structure, do not illustrate the effect of the protein–protein interactions on the structural stability of the helix. It is known that a sequence of Asp and/or Glu residues together can destabilize the helix within the protein, because they are highly charged, and repel each other.^26^,^27^ The forces of repulsion of such residues are stronger than the hydrogen bonding. Moreover, a cluster of Ile residues with their large bulky R groups tends to disrupt the α-helix structure by disrupting the hydrogen bonding within the protein.^28^ Herein, we show for the first time that the helical structure is disrupted according to hydrophobic and electrostatic forces that appear between the amylin aggregate and the insulin B chain.

### Inhibition of amylin aggregation by insulin is restricted

**Fig. 5:**
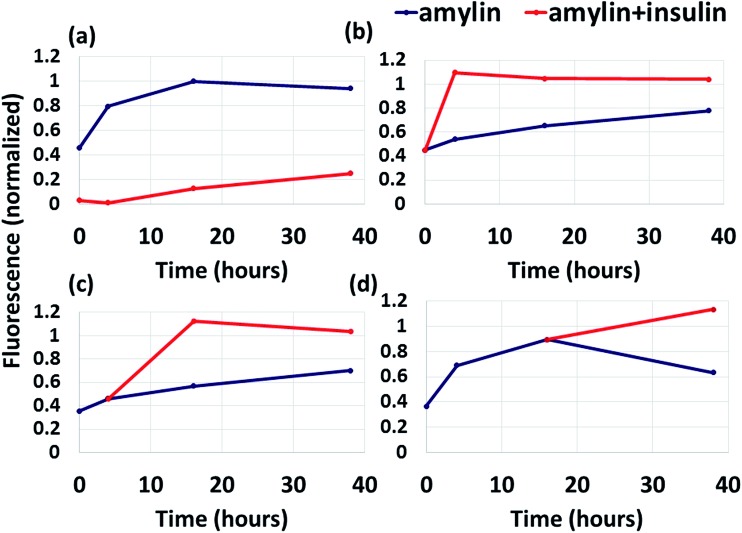
ThT binding fluorescence measurements of the kinetics of amyloid fibril formation. (a) Amylin was added to a buffer already containing insulin. (b) Insulin was added to amylin a few minutes after the dilution of amylin in the buffer. (c) Insulin was added to amylin after 4 hours of incubation (during the growth phase). (d) Insulin was added to amylin after 16 hours of incubation (during the saturation phase).

While previous studies showed that insulin inhibits amylin aggregation,^13^,^17^ but not at long timescales,^17^ it is still unknown what causes the restriction of insulin ability to inhibit amylin aggregation. Fig. 5a shows that the addition of amylin into a solution already containing insulin results in the inhibition of amylin aggregation at short timescale (up to 5 hours), but at longer timescales insulin inhibits amylin aggregation only slightly. Although the ThT binding fluorescence assay shows formation of amylin aggregates in the presence of insulin, it still shows formation of fewer aggregates than in the absence of insulin (Fig. 5a). Herein, we identified for the first time the specific binding site of insulin in amylin aggregates. It could be that the free insulin molecules decreased with time, due to the interaction of insulin molecules with amylin peptides, and therefore the free amylin peptides in the solution have a greater opportunity to interact and produce amylin aggregates. It may also be that the insulin molecules interact among themselves and produce insulin aggregates.

We also found that the interaction between insulin and the amylin aggregate decreases the formation of the β-strand in the C-termini of the structural amylin aggregate of model B1 (Fig. S17†), therefore suggesting that insulin poorly inhibits the formation of amylin aggregates when insulin “is added” to the well-organized fibrillary amylin aggregate. To prove this, insulin was added to amylin in solution in three different states: after dilution of amylin in the buffer, during the growth phase and during the saturation phase (Fig. 5b–d). In all these three states, insulin does not function as an inhibitor for amylin aggregation, but more as a catalyst. Previously it was suggested that at longer timescales (after 72 hours) insulin co-aggregates with amylin,^17^ but it had not been proven that it may appear earlier than 72 hours. Herein we show that the co-aggregation process appears also at shorter timescales than 72 hours of incubation. We therefore suggest that the addition of insulin to amylin aggregates does not inhibit amylin aggregation, but promotes amylin aggregation and may induce co-aggregation between insulin and amylin.

## Conclusions

The effect of amylin on insulin secretion from β-cells has previously been studied,^9^ but the influence of insulin on amylin aggregation remains controversial. While it was proposed that insulin binds to amylin, the molecular mechanisms of the binding sites in the context of full-length insulin and amylin were yet to be studied. In this report, we present for the first time the specific binding site at the molecular level that demonstrates the unique π-stacking between Y16 in insulin and F15 in amylin aggregates. Previous simulation studies of separated amylin fibrils pointed to specific residues along the amylin sequence that are responsible for the stability of amylin fibrils.^29^–^31^ Mutations in other amylin fibrils (*e.g.* rat, pig, and cat) may be relevant for future tests to investigate the interactions with insulin. Moreover, the specific interactions between an insulin molecule and an unstructured amylin monomer or amylin oligomers and their effect on amylin aggregation are still elusive. Future studies are necessary to examine whether these π-stacking interactions between Y16 in insulin and F15 in amylin monomers or oligomers are also present in such systems.

Insulin has several important functions, such as regulation of lipids synthesis, regulation of enzymatic activity and above all regulation of blood glucose levels and prevention of hyperglycemia. Insulin itself can also undergo aggregation in its partially unfolded state.^32^–^35^ *In vitro* studies proposed that the aggregation of insulin results in the deactivation of insulin as a regulator of glucose levels, therefore complicating the therapy for T2D.^36^ Similar to insulin, amylin plays a role in glucose homeostasis, but is found as aggregates in the pancreatic β-cells of type 2 diabetic patients.

**Fig. 6:**
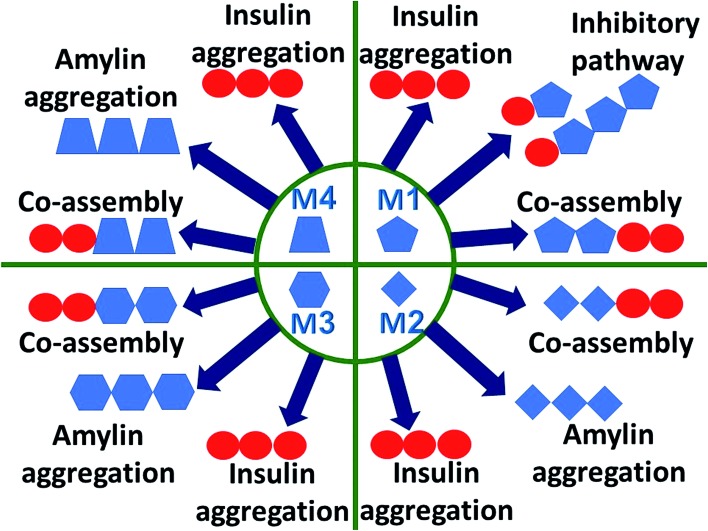
Proposed molecular mechanism co-operation pathways between insulin and amylin. The secretion of amylin from the pancreas in patients with T2D may lead to amylin aggregation. Polymorphic amylin aggregates may be produced (M1–M4). Insulin is more likely to interact with amylin aggregate M1 and to inhibit the amylin aggregation. This inhibitory process is limited, because of the competition between insulin aggregation and cross-seeding between insulin and amylin aggregates (M1–M4). Further processes that may occur in parallel include amylin aggregation of other amylin forms (M2–M4).

Understanding the link between the activities of these two proteins is crucial in gaining insights into the molecular mechanisms of the development of T2D. The question whether amylin monomers could be an inhibitor for insulin aggregation has not been addressed yet. An analog of amylin that mimics the native amylin demonstrated inhibition for insulin aggregation without blocking the functions of insulin.^37^ The current study is aimed at understanding the molecular mechanisms through which insulin interacts with amylin aggregates and not analogs of amylin. The proposed molecular mechanisms are schematically illustrated and summarized in Fig. 6. We propose that insulin can inhibit amylin aggregation by binding to a monomeric amylin. Insulin molecules also reduce amylin aggregation by binding to a specific domain in a specific structure of the amylin aggregate *via* an exothermic process (Fig. S18†). Insulin prefers to interact with a unique amylin aggregate, M1, and has a relatively lower preference for interaction with the other amylin aggregates (models M2–M4). The function of insulin as an inhibitor to amylin aggregation is limited, because of the competition of three processes with the inhibitory function: insulin aggregation, amylin aggregation and co-aggregation between insulin aggregates and amylin aggregates. In summary, in this report we suggest that insulin could be a limited inhibitor for amylin aggregation and in some cases, it plays the opposite function and causes homo- and hetero-amyloid aggregation of insulin and amylin.

## Materials and methods

### Inhibition of amylin by insulin-derived short peptides

Human amylin (Calbiochem Inc., CA, USA) was dissolved in HFIP to produce a 400 μM stock solution. The stock solution was then added to 40 μM insulin B chain derived peptide (Peptron Inc., Taejeon, Korea) solutions in 10 mM sodium acetate buffer pH 6.5 (10-fold excess for peptides), or to 10 mM sodium acetate buffer alone to achieve a final concentration of 4 μM. Immediately after dilution, the samples were centrifuged for 15 min at 20 K rcf at 4 °C, and the pellet was removed. Aliquots of the reaction solutions were diluted 10-fold in a sodium acetate buffer with 0.03 μM thioflavin T (ThT). Fluorescence values were measured immediately after preparation and after 48 and 73 hours, at an excitation of 450 nm and an emission of 480 nm, using a Jobin Yvon Horiba Fluoromax 3 fluorimeter. The experiment was performed with three independent repeats. The average values are presented, with bars (in the column graph) indicating the standard deviations.

### Kinetic measurements of amylin aggregation by the addition of insulin in different aggregation states

Several samples of 4 μM amylin were prepared as described above and incubated for amyloid formation. In addition, a sample was prepared in which amylin was diluted in a buffer already containing human insulin (Sigma). At different time points, one of the samples was divided into two and insulin was added to one of them at a 4 μM concentration. Aggregation was measured using the ThT fluorescence assay described above.

### Molecular modeling

The constructed models of amylin aggregates, insulin and insulin–amylin aggregates are detailed in the ESI appendix.† MD simulations of the constructed models of insulin, amylin aggregates and insulin–amylin aggregates were performed in the NPT ensemble using NAMD^38^ with the CHARMM27 force-field.^39^,^40^ The models were energy minimized and explicitly solvated in a TIP3P water box^41^,^42^ with a minimum distance of 15 Å from each edge of the box. Each water molecule within 2.5 Å of the models was removed. Counter ions were added at random locations to neutralize the charge of the models. The Langevin piston method^38^,^43^,^44^ with a decay period of 100 fs and a damping time of 50 fs was used to maintain a constant pressure of 1 atm. The temperature, 330 K, was controlled using a Langevin thermostat with a damping coefficient of 10 ps.^38^ In order to examine the stability of the constructed models, higher temperature (330 K) than the physiological temperature (300 K) has been applied in the current work. It is expected that if the constructed models are stable at this temperature then these constructed models are also stable at physiological temperature. A Langevin damping coefficient value of 10 ps has been chosen as an optimal parameter to keep the temperature reasonably constant. The short-range van der Waals (VDW) interactions were calculated using the switching function, with a twin range cutoff of 10.0 and 12.0 Å. Long-range electrostatic interactions were calculated using the particle mesh Ewald method with a cutoff of 12.0 Å.^45^,^46^ The equations of motion were integrated using a leapfrog integrator with a time step of 1 fs.

The solvated systems were energy minimized for 2000 conjugated gradient steps, where the hydrogen bonding distance between the β-sheets in the amylin aggregates is fixed in the range 2.2–2.5 Å. The counter ions and water molecules were allowed to move. The hydrogen atoms were constrained to the equilibrium bond using the SHAKE algorithm.^47^ The minimized solvated systems were energy minimized for 5000 additional conjugate gradient steps and 20 000 heating steps at 250 K, with all atoms allowed to move. Then, the systems were heated from 250 K to 300 K and then to 330 K for 300 ps and equilibrated at 330 K for 300 ps. These conditions were applied to all variant models. Simulations were run for 100 ns for each variant model. The models that were simulated include four amylin fibrillary models, an insulin molecule and sixteen insulin–amylin fibrillary models. Therefore, a total run time of 2.1 μs for all variant models was employed. Table S2† summarizes all simulated models. These timescales of simulations were chosen after examining the convergence of the simulated models, using hydrogen bond analysis and root mean square deviation (RMSD) analysis (ESI appendix, Fig. S19–S21†). The simulated structural models were saved every 10 ps for analysis.

### Data analysis

The analysis of the conformational energies and populations of the simulated models is illustrated in detail in the ESI appendix.† The structural stabilities of the studied models were examined by following the changes in the number of hydrogen bonds between β-strands, with the hydrogen bond cut-off being set to 2.5 Å. This examination was performed also by following the RMSDs of all of the examined structures. The hydrophobic, electrostatic and π–π interactions were determined by measuring the distances between atoms (ESI appendix†). To estimate the secondary structure of the self-assembled models the hydrogen bond estimation algorithm Define Secondary Structure of Proteins (DSSP) was applied in the last 5 ns of the simulation for each studied model.

## Conflicts of interest

There are no conflicts to declare.

## Supplementary Material

Supplementary information
