# Supplementary material for: Mechanistic perspective and functional activity of insulin in amylin aggregation
Source: Chem Sci. 2018 Apr 16;9(18):4244–52. doi: 10.1039/c8sc00481a (PMC5944211; doi:10.1039/c8sc00481a)
Supplement: Supplementary file 1 [file SC-009-C8SC00481A-s001.pdf]

Electronic Supplementary Material (ESI) for Chemical Science.
This journal is © The Royal Society of Chemistry 2018

Supporting Information for

"Mechanistic perspective and functional activity of insulin on amylin aggregation"

Michal Baram,[1,2] Sharon Gilead,[3] Ehud Gazit[3,4,*] and Yifat Miller[1,2,*]

[1]Department of Chemistry Ben-Gurion University of the Negev, Be'er Sheva 84105, Israel
[2]The Ilse Katz Institute for Nanoscale Science & Technology Ben-Gurion University of the Negev, Be'er Sheva 84105, Israel
[3]Department of Molecular Microbiology and Biotechnology, Tel Aviv University, Tel Aviv 69978, Israel
[4]Department of Materials Science and Engineering, Iby and Aladar Fleischman Faculty of Engineering, Tel Aviv University, Tel Aviv 69978, Israel

Corresponding authors:

Yifat Miller: ymiller@bgu.ac.il

Ehud Gazit: ehudg@post.tau.ac.il

Inhibition of amylin with insulin B chain derived peptides (alanine scan)

Inhibition was measured using the ThT fluorescence assay as described in the materials and methods. Human amylin (Calbiochem Inc., CA, USA) was dissolved in HFIP to produce a 400 μm stock solution. The stock solution was then distributed into solutions containing 40 μm inhibiting peptides (Peptron Inc., Taejeon, Korea) in 10 mM sodium acetate buffer pH 6.5 (10-fold excess for inhibiting peptides), or into 10 mM sodium acetate buffer alone to a final concentration of 4 μM. Immediately after dilution, the samples were centrifuged for 15 min at 20K rcf at 4 ºC, and the pellet was removed. Aliquots of the reaction solutions were diluted 10-fold into a sodium acetate buffer with 0.03 μM ThT. Fluorescence values were measured immediately after preparation and after different incubation periods, at excitation of 450 nm and an emission of 480 nm, using a Jobin Yvon Horiba Fluoromax 3 fluorimeter. The experiment was performed with three independent repeats. Average values are presented, bars (in column graph) indicating the standard deviations.

Construction of models of insulin-amylin aggregates

Four polymorphic models of amylin hexamers which previously studied by our group[1] were constructed. Figure S1 illustrates these four structural of models of amylin aggregates (M1-M4). These four models illustrate single layer conformations. The experimental structural models of amylin demonstrate double layer conformations, i.e. "protofilaments".[2, 3] The current work focuses only on the single layer conformations. The C-termini of the amylin monomers within the fibrillary structures are non-amidated. Experimental studies showed that the non-amidated at the C-terminal of amylin are known to aggregate slower than the amidated amylin.[4] Herein, we do not investigate the kinetics of amylin aggregation.

The insulin molecule that has been applied in the current study was taken from the crystal structure, pdb id code: 1GUJ.[5]

Sixteen different models of insulin-amylin aggregates were constructed. In each model an insulin molecule (pdb id code: 1GUJ)[5] interacts with each one of the four polymorphic structural amylin hexamers.[1] In the current work, interactions of insulin with double layer conformations have not been investigated. In the case where insulin interacts with the N-termini of amylin's fibrillary domains, one can expect that similar scenario and effects that are seen in the amylin single layer conformations will be

obtained also in the double layer conformations. However, in the case in which insulin interacts with the C-termini of amylin's fibrillary domains in the single layer conformations, insulin cannot interact in the double layer conformation. Therefore, the later case cannot be examined in the double layer conformations.

The binding site recognition domain in which an insulin molecule interacts with amylin peptides have been proposed by two experimental studies. One study suggested that the central domain of insulin B chain (residues 9-20) binds to the first β-strand domain in amylin (residues 7-19).[6] The second study proposed that the C-terminal of insulin B chain (residues 22-29) bind to the second β-strand domain in amylin (residues 23-30).[7] Each one of these two propose recognition domains was examined for each one of the four polymorphic amylin aggregates while binding to the central monomer along the fibrillary structure of amylin aggregate in two different orientations within the recognition domains (as illustrated schematically in Figures S2 and S3).

In Figure S2, insulin chain B (residue 9-20) interact with amylin aggregate in two different orientations within the recognition domain in amylin aggregate: the first orientation is with residues 7-19, and the second with residues 19-7. We modeled eight constructed models of insulin-amylin aggregates: models A1-A4 (Figure S4) and B1-B4 (Figure S5).

In Figure S3, insulin chain B (residue 22-29) interact with amylin aggregate in two different orientations within the recognition domain in amylin aggregate: the first orientation is with residues 23-30, and the second with residues 30-23. We modeled eight constructed models of insulin-amylin aggregates: models C1-C4 (Figure S6) and D1-D4 (Figure S7). It should be noted, that in mode D1 the insulin was escaped from the amylin aggregate during the molecular dynamics (MD) simulations, therefore this model is neglected in this framework.

Finally, mutations of Y16 to A16 in insulin molecule were performed for models A1 and B1, using the original initial constructed models – producing models P1 and Q1, respectively.

Determining the Conformational Energies and Populations for the Simulated Models of Insulin-Amylin Aggregates

To obtain the relative structural stability of the variant models, the trajectories of the last 5 ns were first extracted from the explicit MD simulation excluding water

molecules. The solvation energies of all systems were calculated using the Generalized Born Method with Molecular Volume (GBMV).[8, 9] In the GBMV calculations, the dielectric constant of water was set to 80.0. The hydrophobic solvent-accessible surface area (SASA) term factor was set to 0.00592 kcal/mol·$Å^2$. Each variant is minimized 1000 cycles and the conformation energy is evaluated by grid-based GBMV. The minimization does not change the conformations of each variant, but only relaxed the local geometries due to thermal fluctuation which occurred during the MD simulations.

A total of 8,000 conformations (500 conformations for each of the 16 examined conformers) were used to construct the free energy landscape of the conformers and to evaluate the conformer probabilities by using Monte Carlo (MC) simulations. In the first step, one conformation of conformer i and one conformation of conformer j were randomly selected. Then, the Boltzmann factor was computed as e-(Ej-Ei)/kT, where Ei and Ej are the conformational energies evaluated using the GBMV calculations for conformations i and j, respectively, k is the Boltzmann constant and T is the absolute temperature (298 K used here). If the value of the Boltzmann factor was larger than the random number, then the move from conformation i to conformation j was allowed. After 1 million steps, the conformations 'visited' for each conformer were counted. Finally, the relative probability of model n was evaluated as Pn= Nn/Ntotal, where Pn is the population of model n, Nn is the total number of conformations visited for model n, and Ntotal is the total steps. The advantages of using MC simulations to estimate conformer probability lie in their good numerical stability and the control that they allow of transition probabilities among several conformers .

Using all twelve models and 8,000 conformations (500 for each model) generated from the MD simulations, we estimated the overall stability and populations for each conformer based on the MD simulations, with the energy landscape being computed with GBMV for these twelve models. The group of these twelve models is likely to present may be only a very small percentage of the ensemble. Nevertheless, the carefully selected models cover the most likely structures.

To validate the choice of the statistical calculations of 500 conformations for each model, we applied also statistical calculations of 1000 conformations from the last 10 ns for each model. One can see from Figures S22-S24, that the energies are similar and the relative conformational energies illustrate similar trends.

Determining hydrophobic, electrostatic and π-π interactions

The hydrophobic interactions between two residues were estimated by measurements of the distances between Cα atoms of two residues. The cutoff distance for hydrophobic interactions is 10 Å.[10] The electrostatic interactions between two residues were estimated by the distance measurements between O atom of the acidic residue and the N atom of the base residue. The cutoff distance for electrostatic interactions is 4 Å.[11] The cutoff distance between C atom of two aromatic residue that estimated the π-π interactions is 7 Å.[12] The specific C atoms of the aromatic residues of each model are summarized in Table S1.

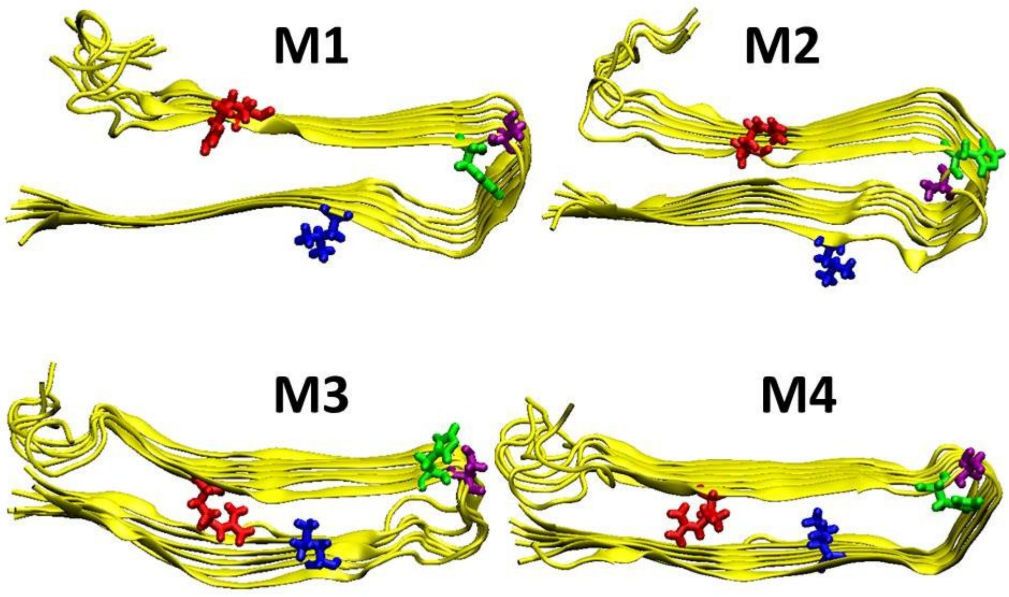

Figure S1: Initial constructed models M1-M4 of amylin hexamer, adopted from Wineman-Fisher et al.[1] These models are based on Tycko's ssNMR structures[2] and on Eisenberg's crystal structures.[3]

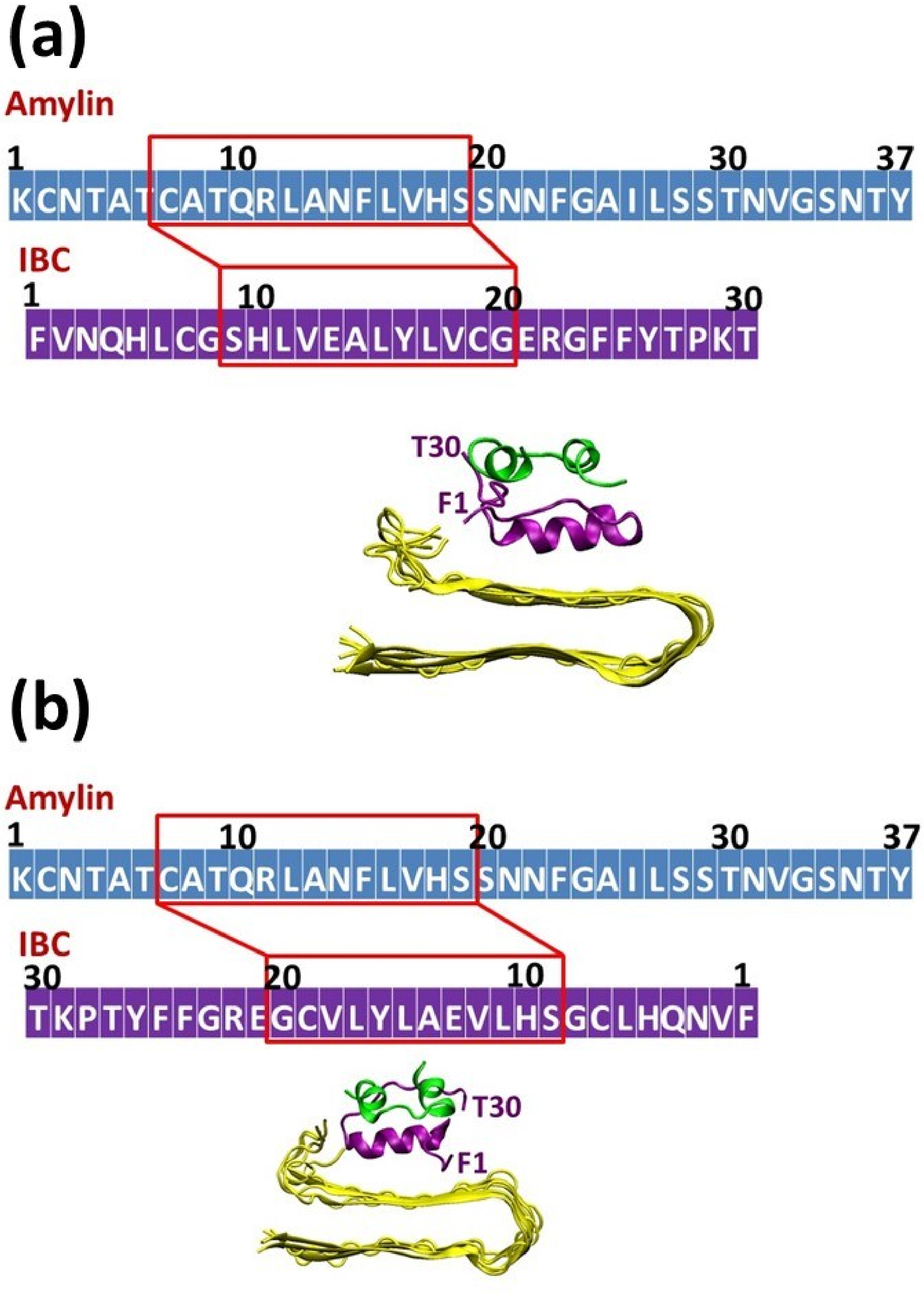

Figure S2: Recognition motif between amylin and insulin B chain (IBC) that was proposed by Gazit group:[6] amylin (7-19) may interacts with (a) IBC (9-20) to produce models A1-A4 (Figure S4) and with (b) IBC (20-9) to produce models B1-B4 (Figure S5).

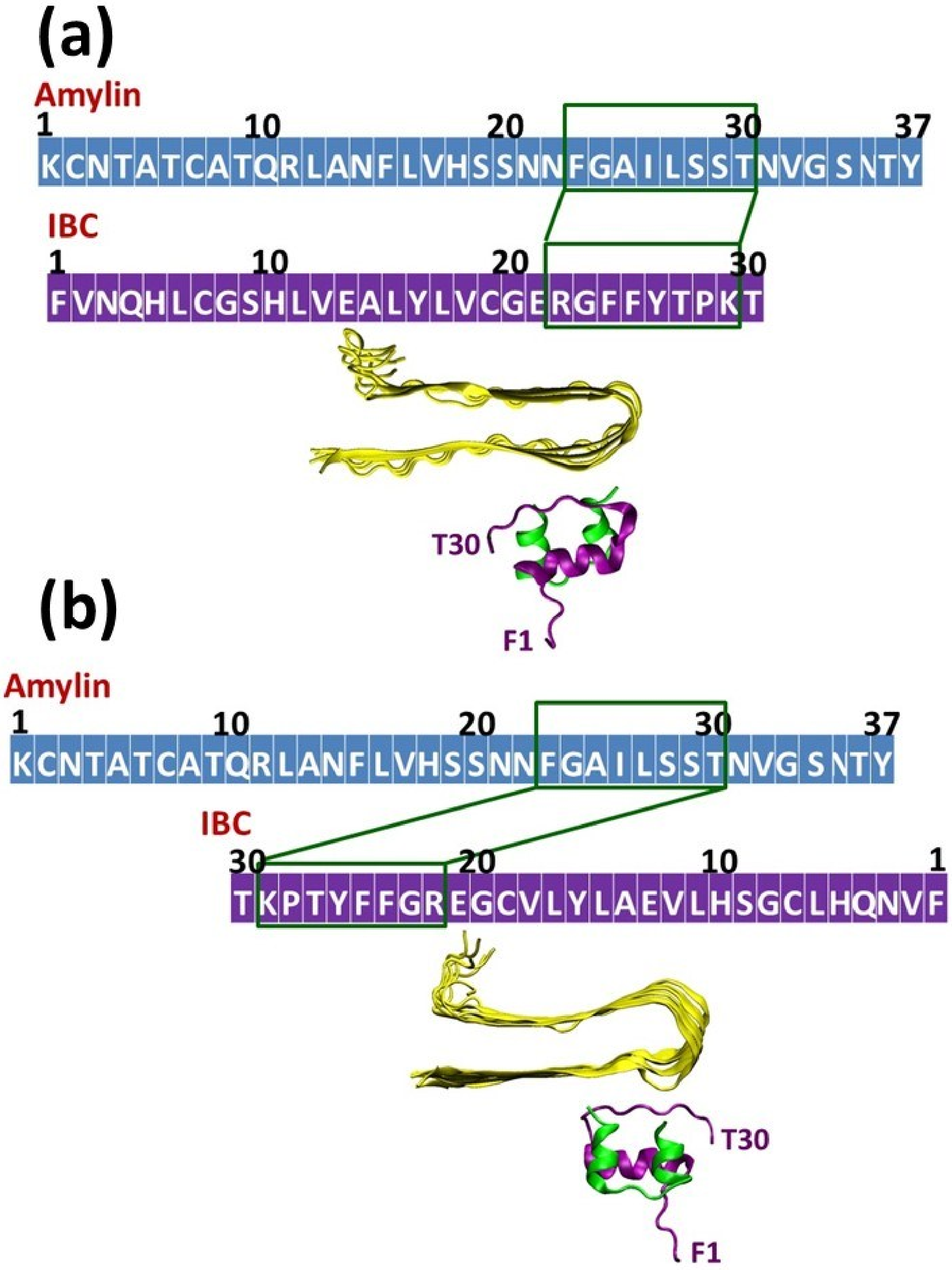

Figure S3: Recognition motif between amylin and insulin B chain (IBC) that was proposed by group:[13] amylin (23-30) may interacts with (a) IBC (22-29) to produce models C1-C4 (Figure S6) and with (b) IBC (29-22) to produce models D1-D4 (Figure S7).

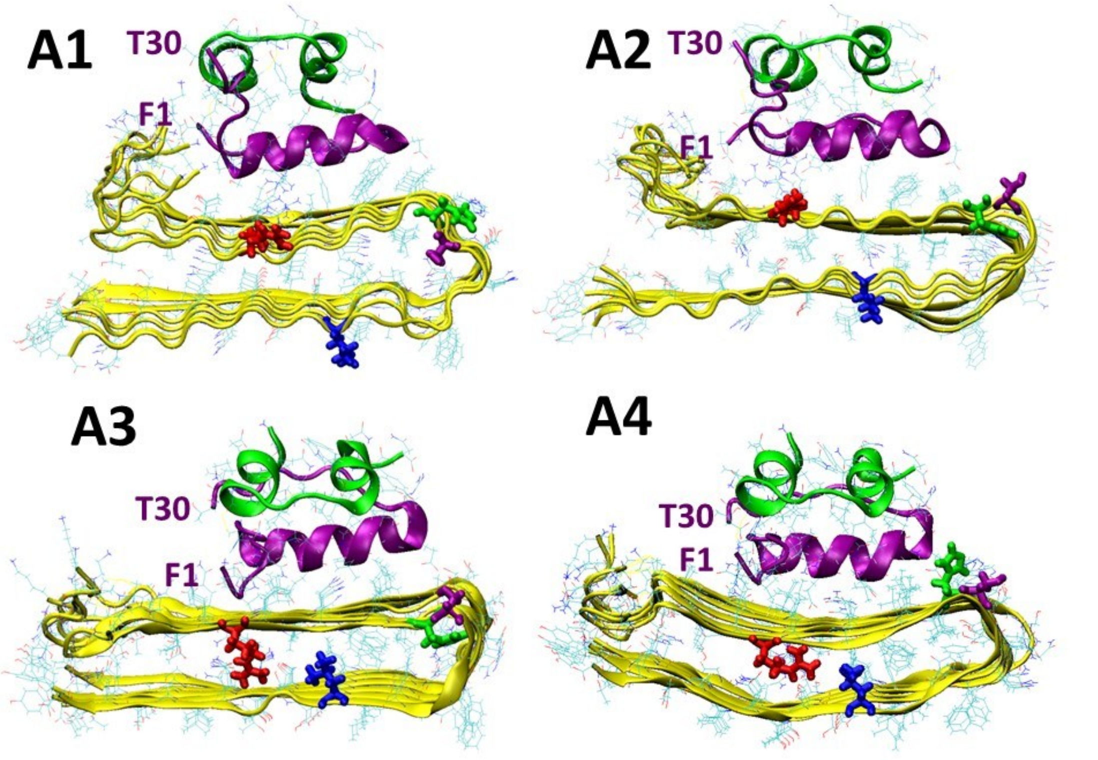

Figure S4: Initial constructed models of insulin-amylin aggregates. The interaction interfaces between insulin (1GUJ)[5] and each of the four amylin aggregates (Figure S1) were constructed according to proposed recognition motif as illustrated schematically in Figure S2a.

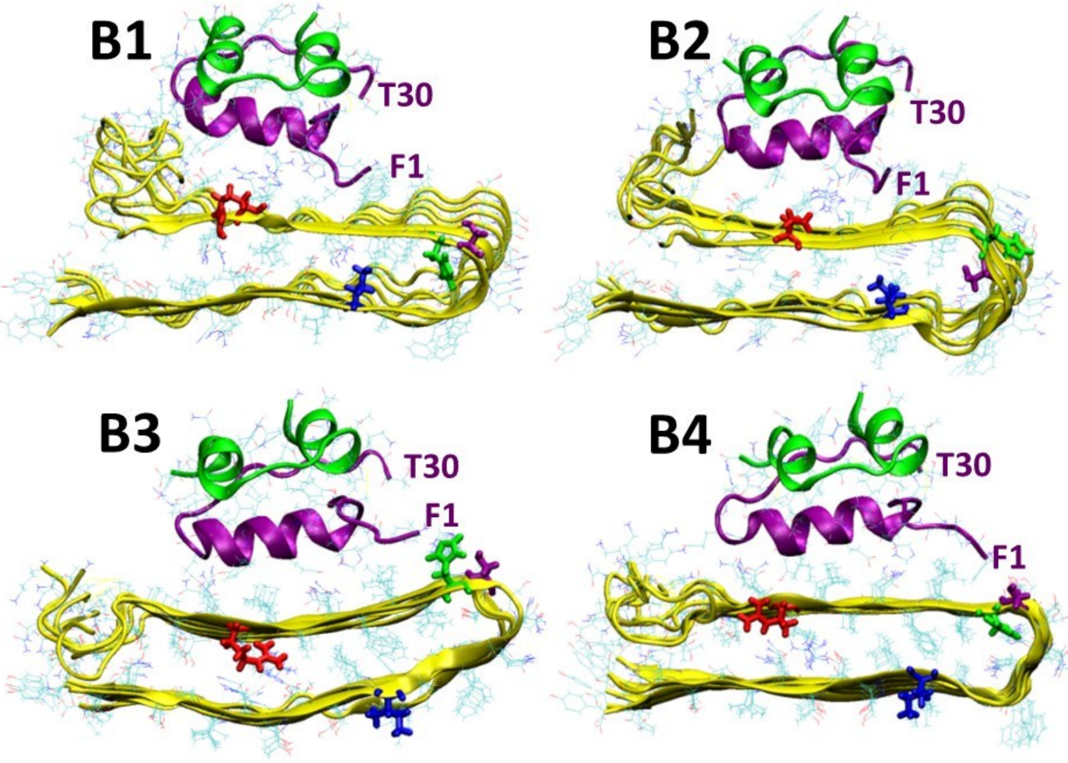

Figure S5: Initial constructed models of insulin-amylin aggregates. The interaction interfaces between insulin (1GUJ)[5] and each of the four amylin aggregates (Figure S1) were constructed according to proposed recognition motif as illustrated schematically in Figure S2b.

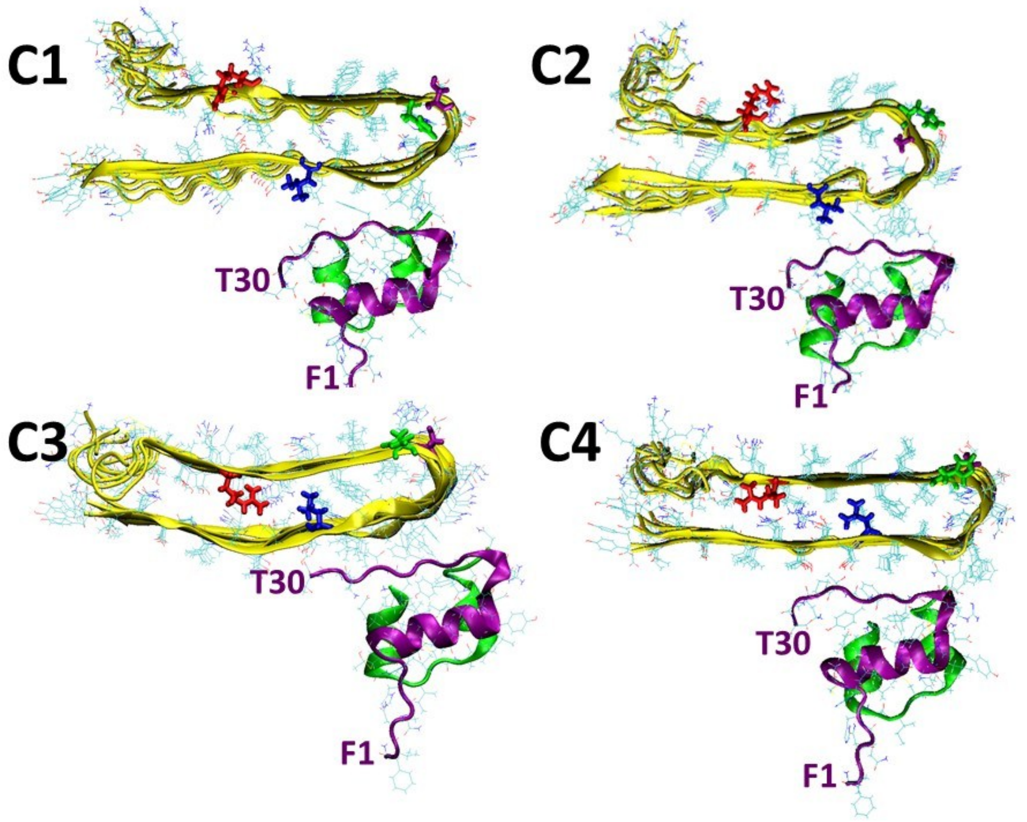

Figure S6: Initial constructed models of insulin-amylin aggregates. The interaction interfaces between insulin (1GUJ)[5] and each of the four amylin aggregates (Figure S1) were constructed according to proposed recognition motif as illustrated schematically in Figure S3a.

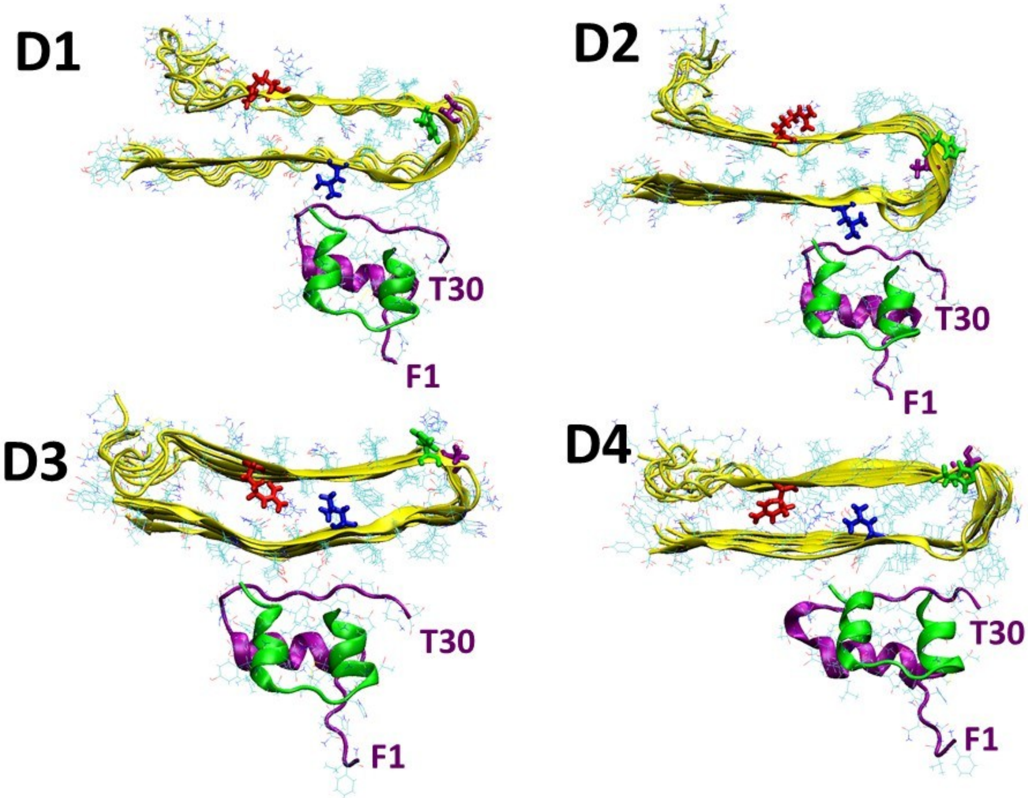

Figure S7: Initial constructed models of insulin-amylin aggregates. The interaction interfaces between insulin (1GUJ)[5] and each of the four amylin aggregates (Figure S1) were constructed according to proposed recognition motif as illustrated schematically in Figure S3b.

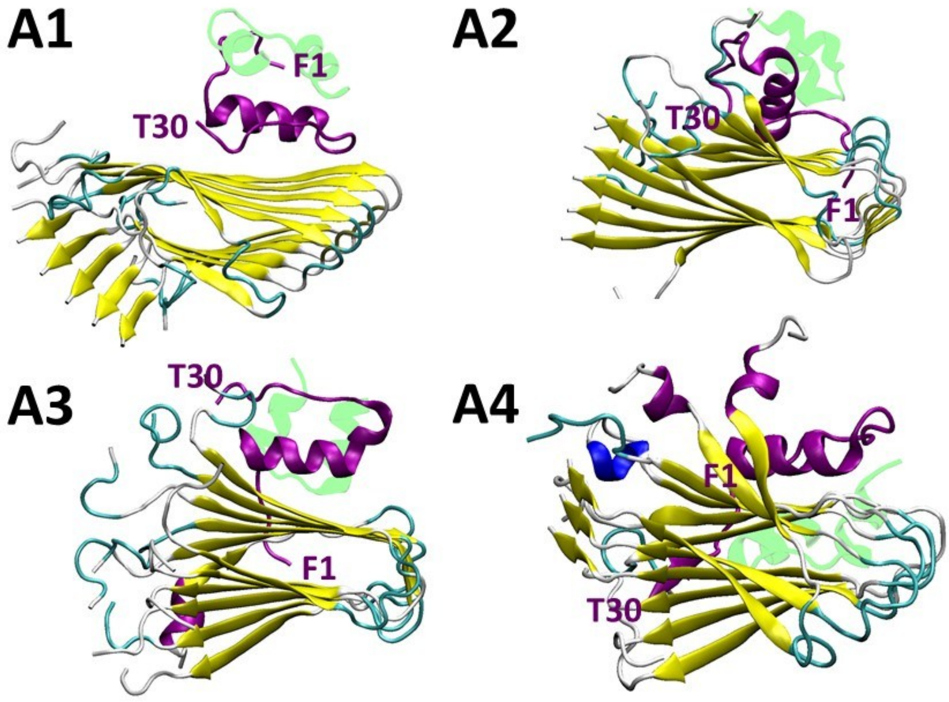

Figure S8: Simulated final structural models of insulin-amylin aggregates of the initial constructed models seen in Figure S4.

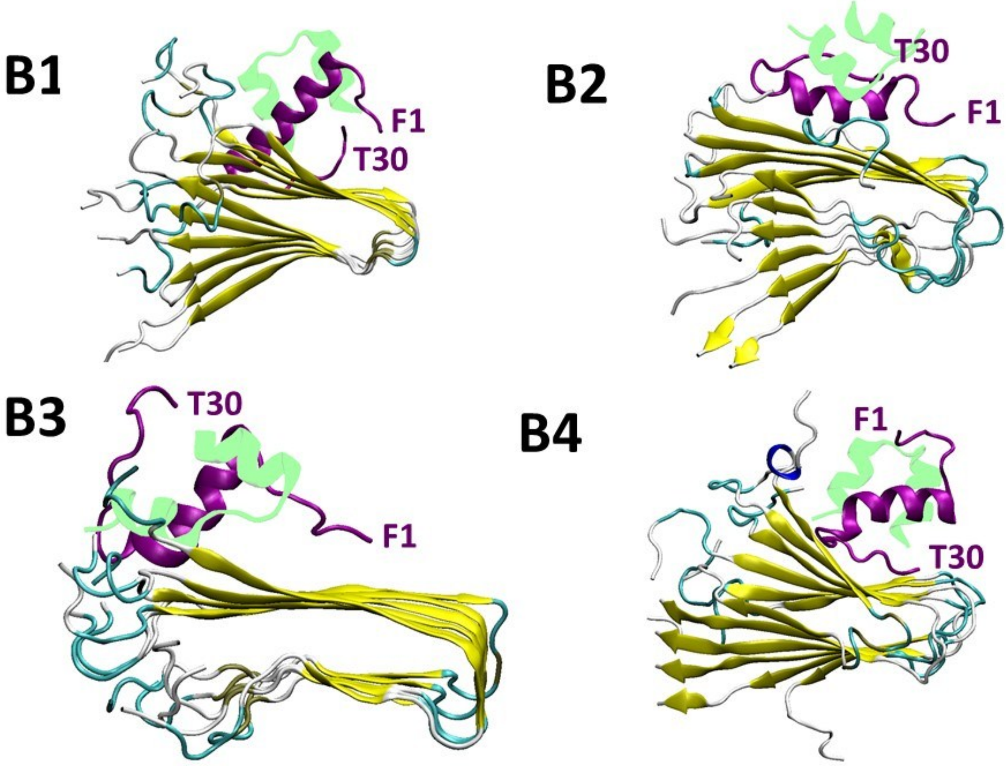

Figure S9: Simulated final structural models of insulin-amylin aggregates of the initial constructed models seen in Figure S5.

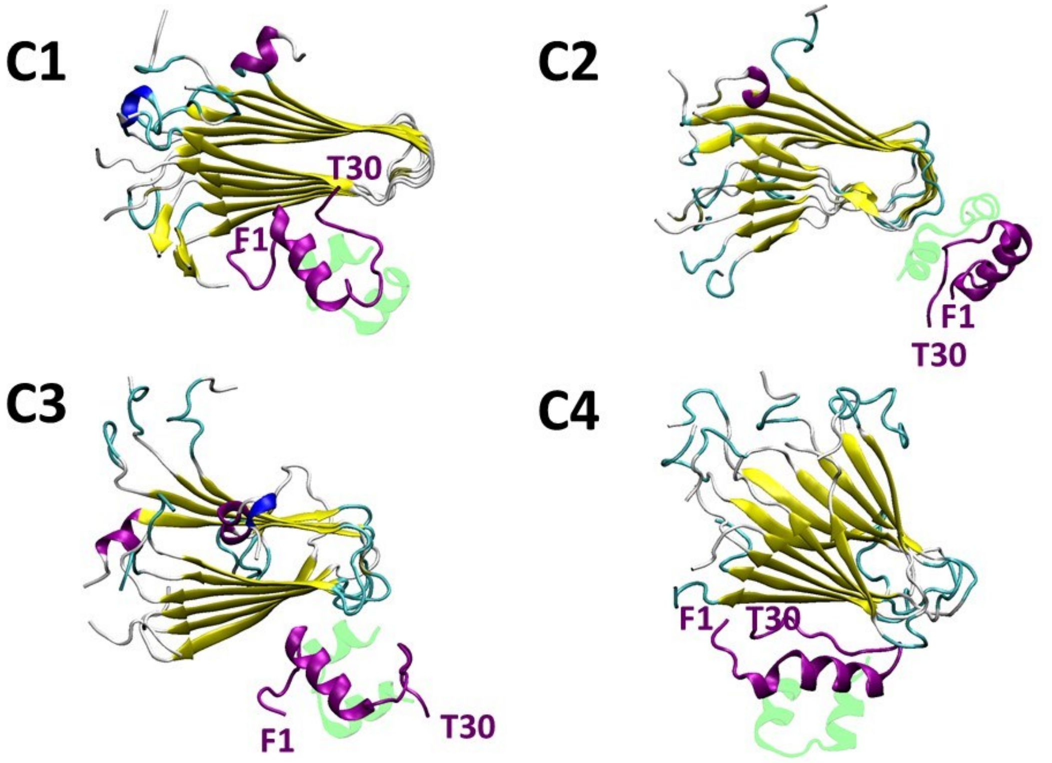

Figure S10: Simulated final structural models of insulin-amylin aggregates of the initial constructed models seen in Figure S6.

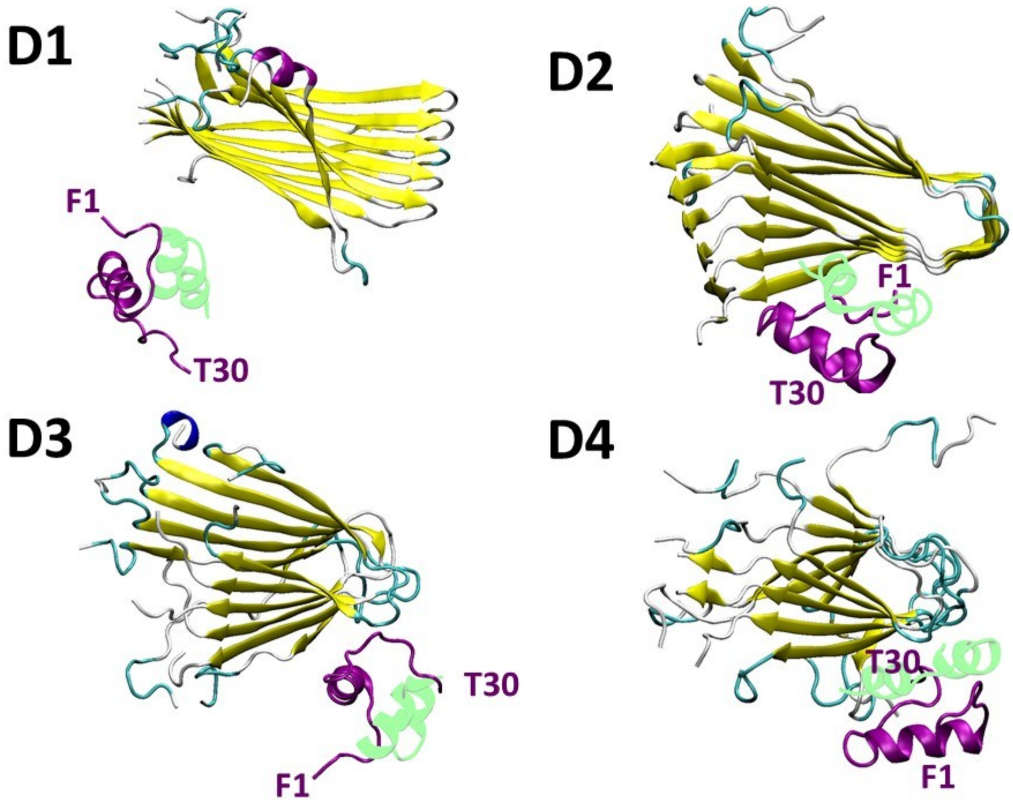

Figure S11: Simulated final structural models of insulin-amylin aggregates of the initial constructed models seen in Figure S7. In model D1, the insulin has been escaped from the amylin aggregate, breaking the insulin-amylin aggregate complex.

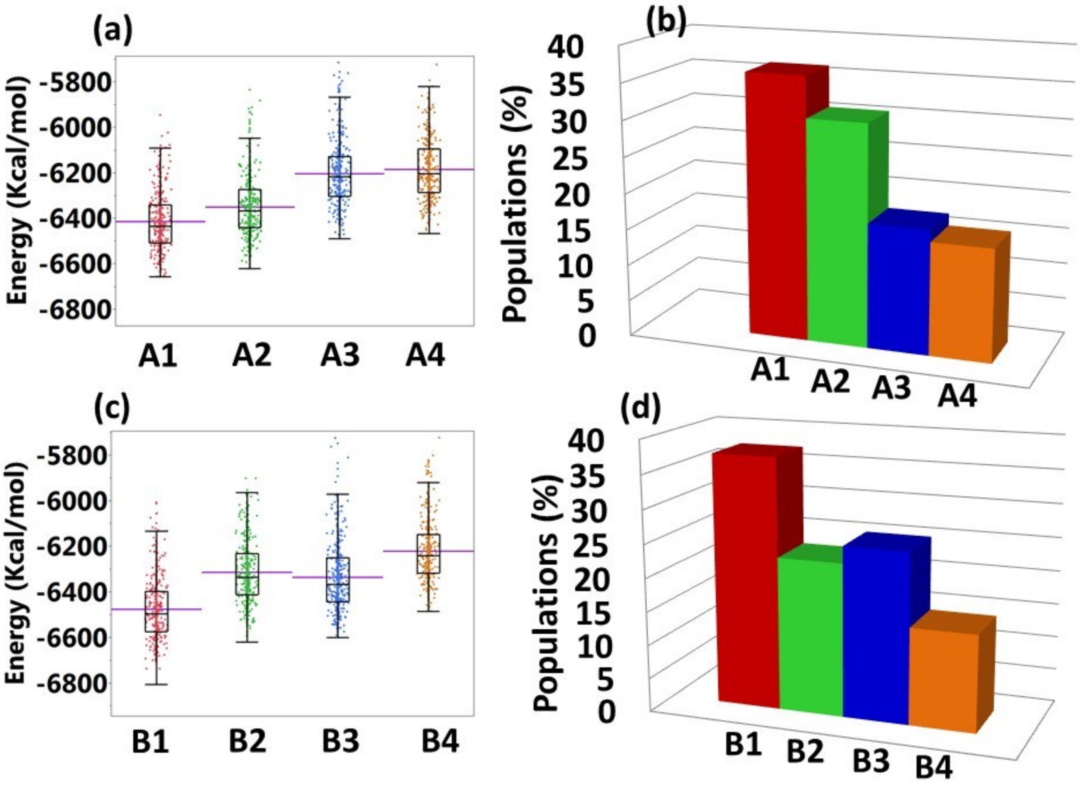

Figure S12: Scatter charts of the 500 conformations obtained from the GBMV energy values extracted from the last 5 ns of each model (a) A1-A4, and (c) B1 and B4. The scatter charts represent the "histograms" of the number of conformations in energies' range. The averaged energy values are seen in the "boxes". The P values for models A1-A4: $P_{A1,A2}$= 1.657x10^{-12}, $P_{A2,A3}$= 2.43x10^{-48}, $P_{A3,A4}$= 0.144; The P values for models B1-B4: $P_{B1,B2}$= 4.97x10^{-56}, $P_{A2,A3}$= 8.10x10^{-4}, $P_{A3,A4}$= 8.52x10^{-37}. (b) and (d) Populations analysis of models A1-A4 and B1-B4.

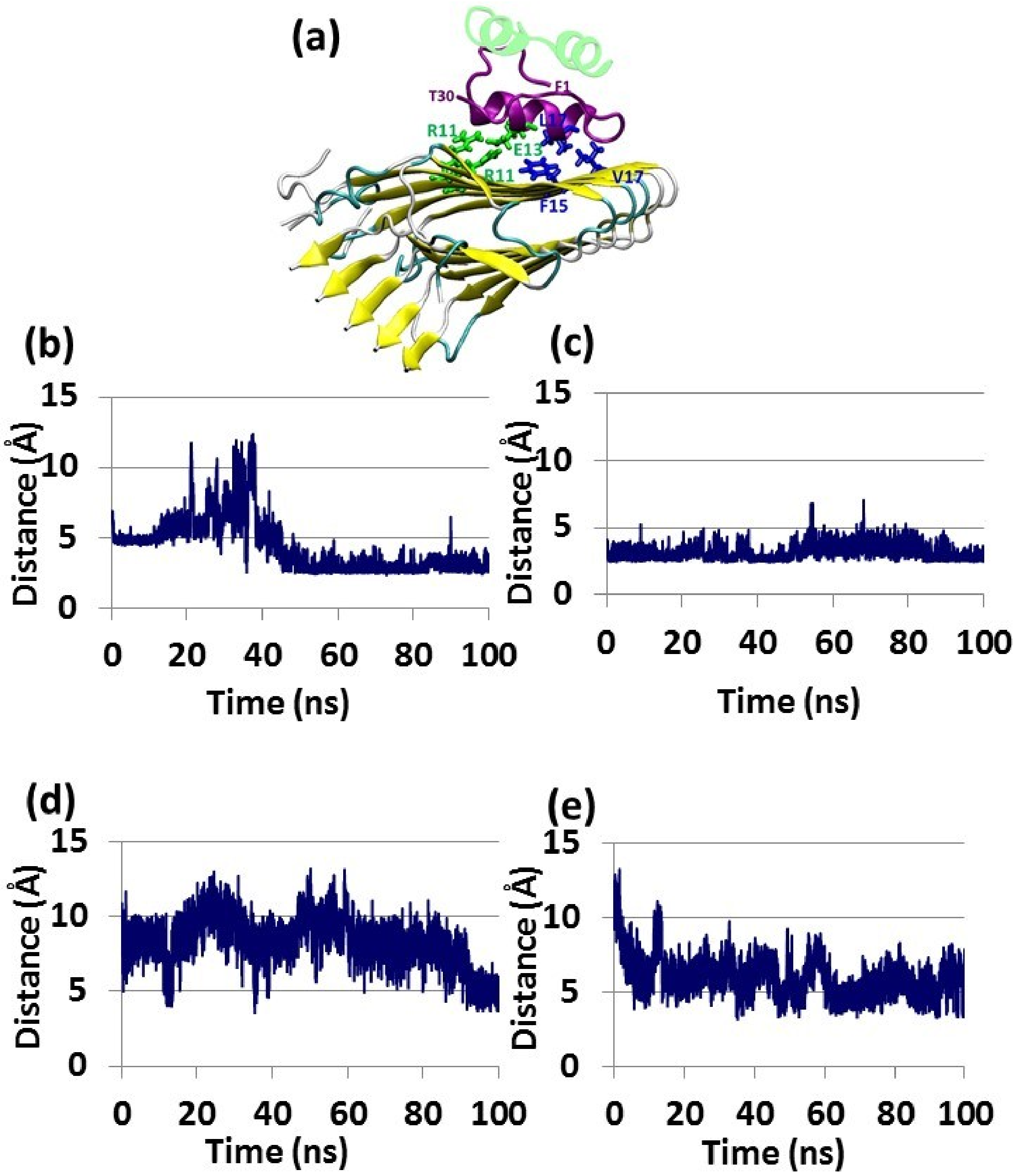

Figure S13: (a) Electrostatic interactions (green) and hydrophobic interactions (blue) in model A1. (b) Distance between R11 in monomer 4 of amylin aggregate and E13 in IBC along the MD simulations. (c) Distance between R11 in monomer 5 of amylin aggregate and E13 in IBC along the MD simulations. (d) Distance between F15 in monomer 6 of amylin aggregate and L17 in IBC along the MD simulations. (e) Distance between V17 in monomer 6 of amylin aggregate and L17 in IBC along the MD simulations.

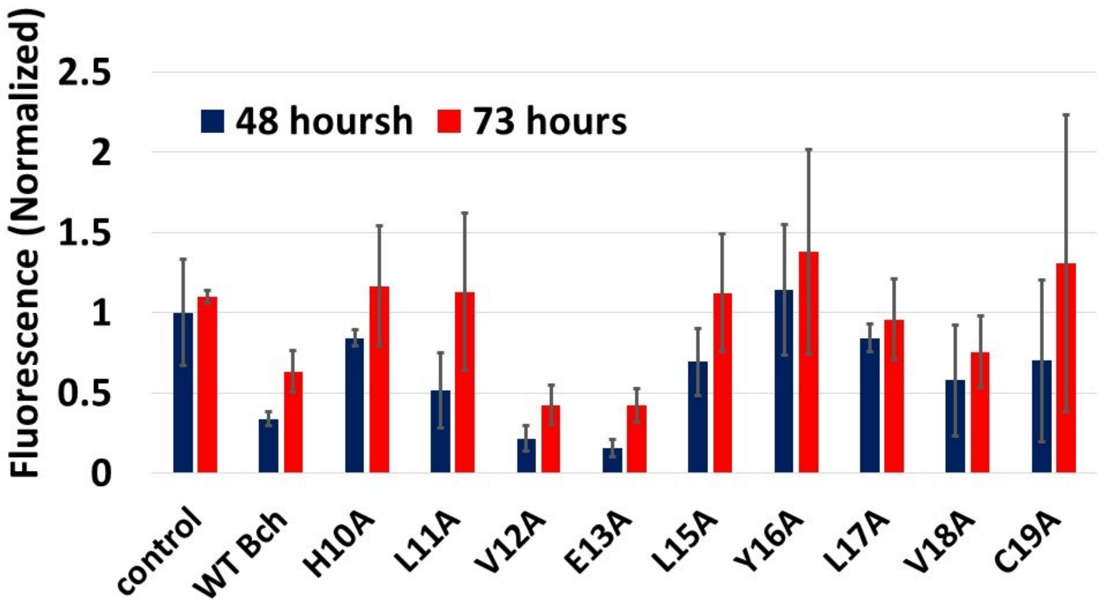

Figure S14: Alanine scan of insulin B chain derived peptide fragments ^{10}HLEVALYV^{19}C. Peptide sequences corresponding to wild-type or single alanine modified fragment of insulin B chain in the recognition domain. The figure illustrates the effect of insulin B chain peptide fragments on amylin fibrillization. ThT fluorescence measurements of amyloid formation by amylin alone (=control), in the presence of a peptide derived of amylin-binding region within insulin B chain (=wt Bch) and in the presence of mutated forms if this peptide (each of the amino acid was substituted with alanine, the numbers represent the position within the B chain). Error bars represent standard deviations of three independent repeats.

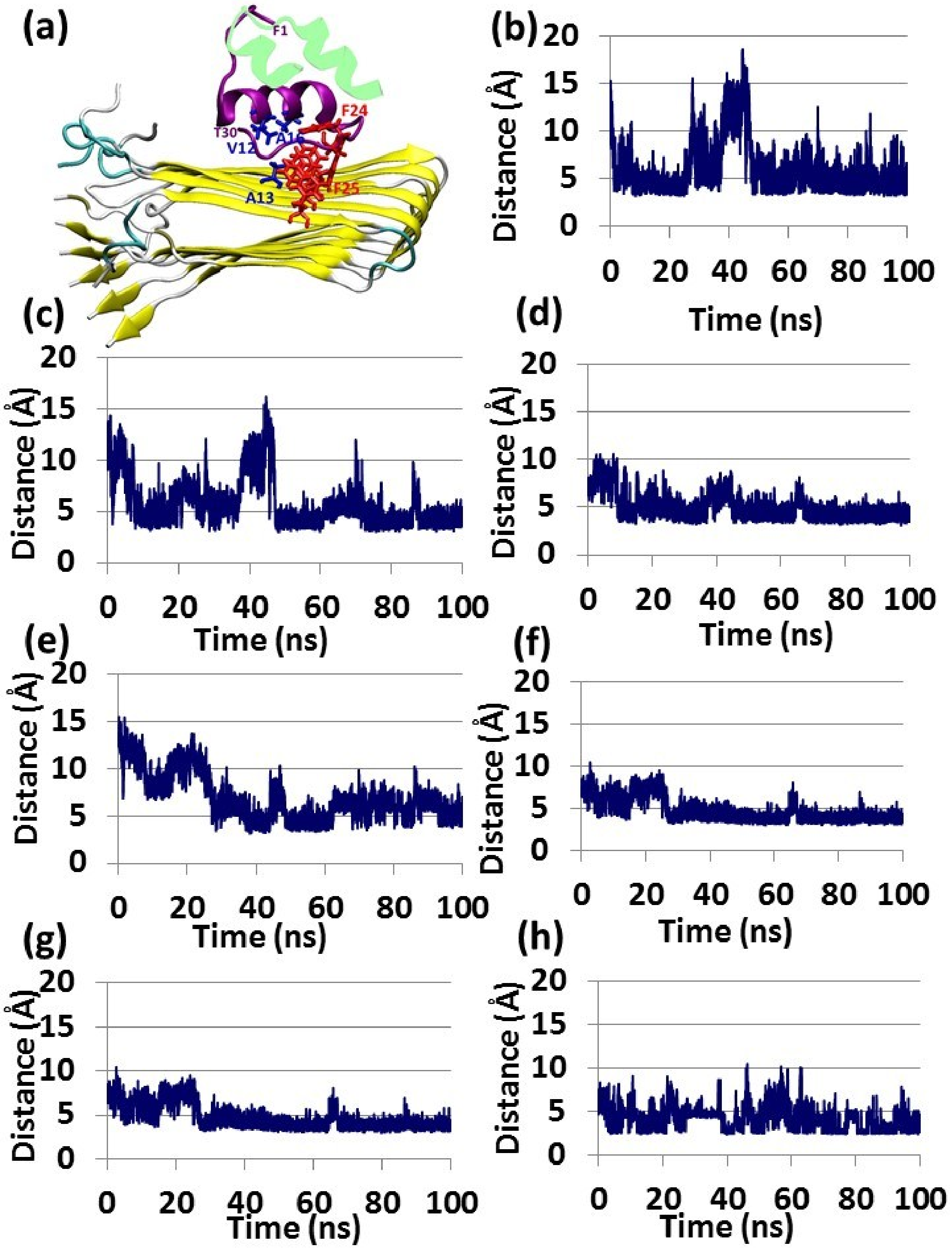

Figure S15: (a) Hydrophobic interactions (blue) and π-π interactions (red) in model P1. (b) Distance between F15 in monomer 1 of amylin aggregate and F25 in IBC along the MD simulations. (c) Distance between F15 in monomer 2 of amylin aggregate and F25 in IBC along the MD simulations. (d) Distance between F15 in monomer 3 of amylin aggregate and F24 in IBC along the MD simulations. (e) Distance between F15 in monomer 3 of amylin aggregate and F25 in IBC along the MD simulations. (f) Distance between F15 in monomer 4 of amylin aggregate and F24 in IBC along the MD simulations. (g) Distance between F15 in monomer 5 of amylin aggregate and A16 in IBC along the MD simulations.

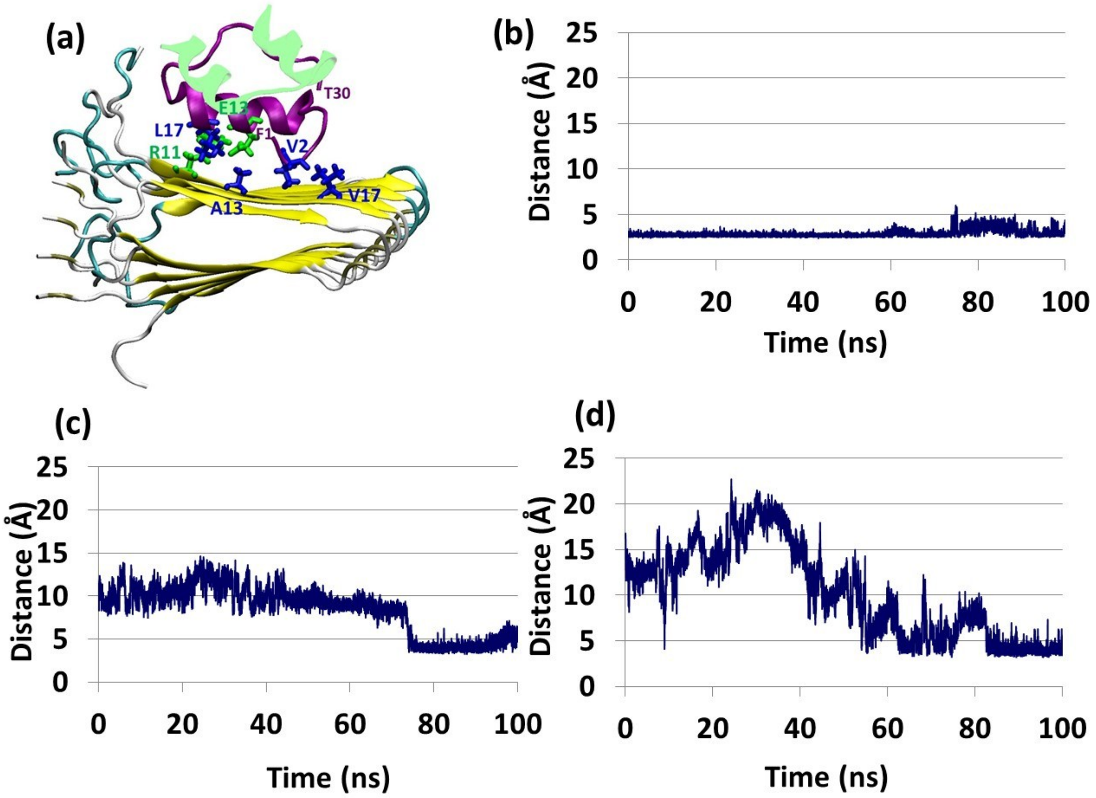

Figure S16: (a) Electrostatic interactions (green) and hydrophobic interactions (blue) in model A1. (b) Distance between R11 in monomer 3 of amylin aggregate and E13 in IBC along the MD simulations. (c) Distance between A13 in monomer 3 of amylin aggregate and L17 in IBC along the MD simulations. (d) Distance between V17 in monomer 3 of amylin aggregate and V2 in IBC along the MD simulations.

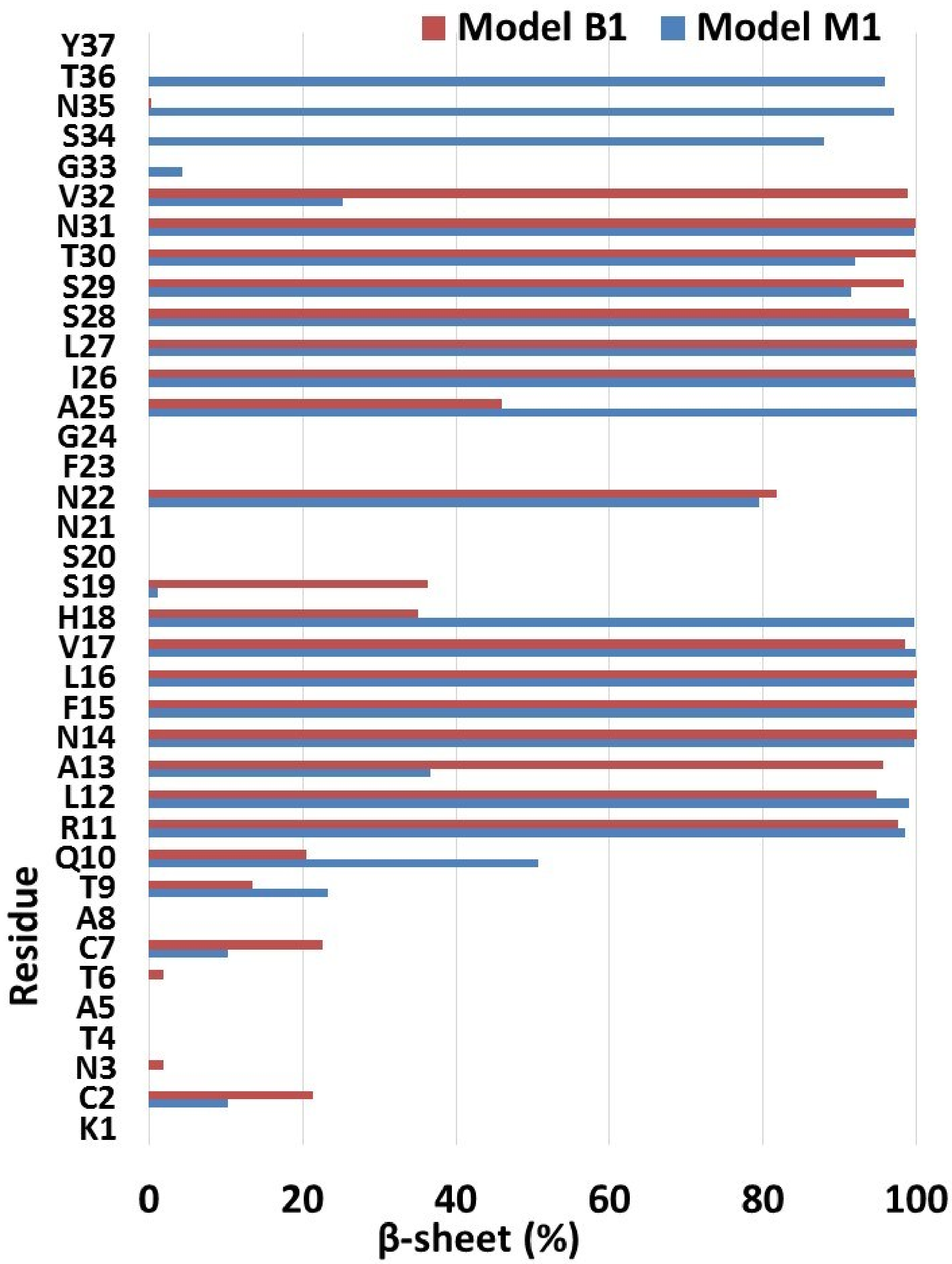

Figure S17: The percentage of β-strand properties of residues along the sequence of amylin in amylin aggregate of model M1 and in insulin-amylin aggregate of model B1.

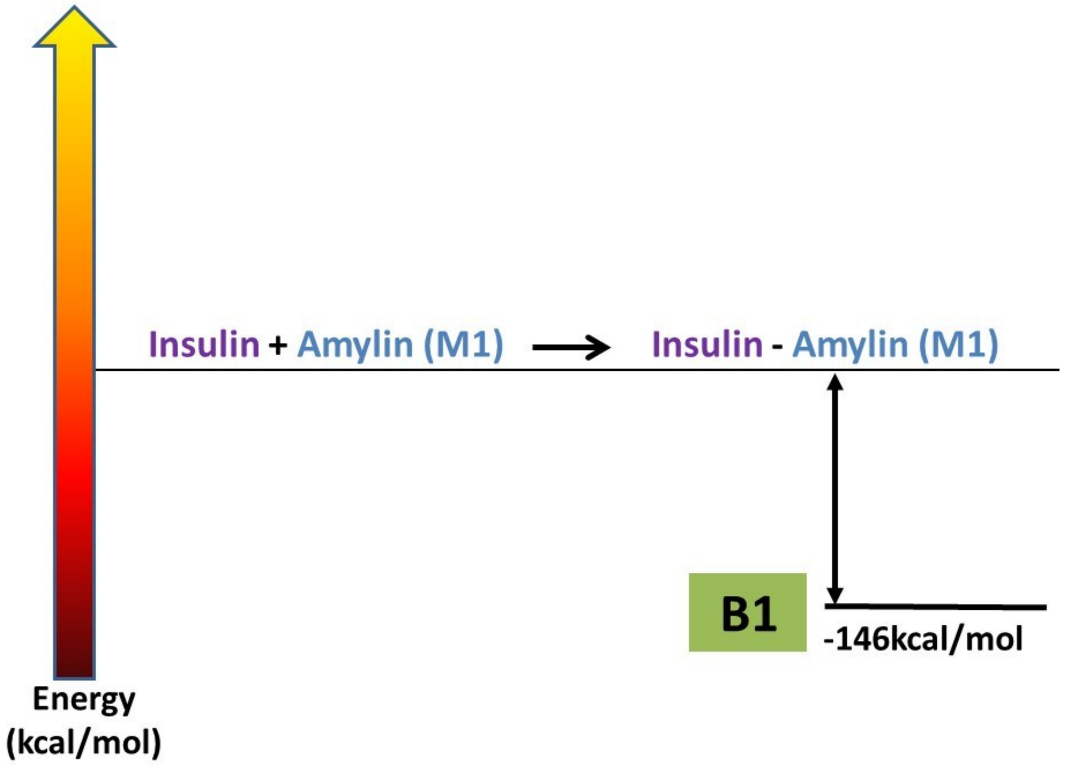

Figure S18: The relative conformational energies of separated insulin and amylin aggregate model M1 and insulin-amylin aggregate complex – model B1.

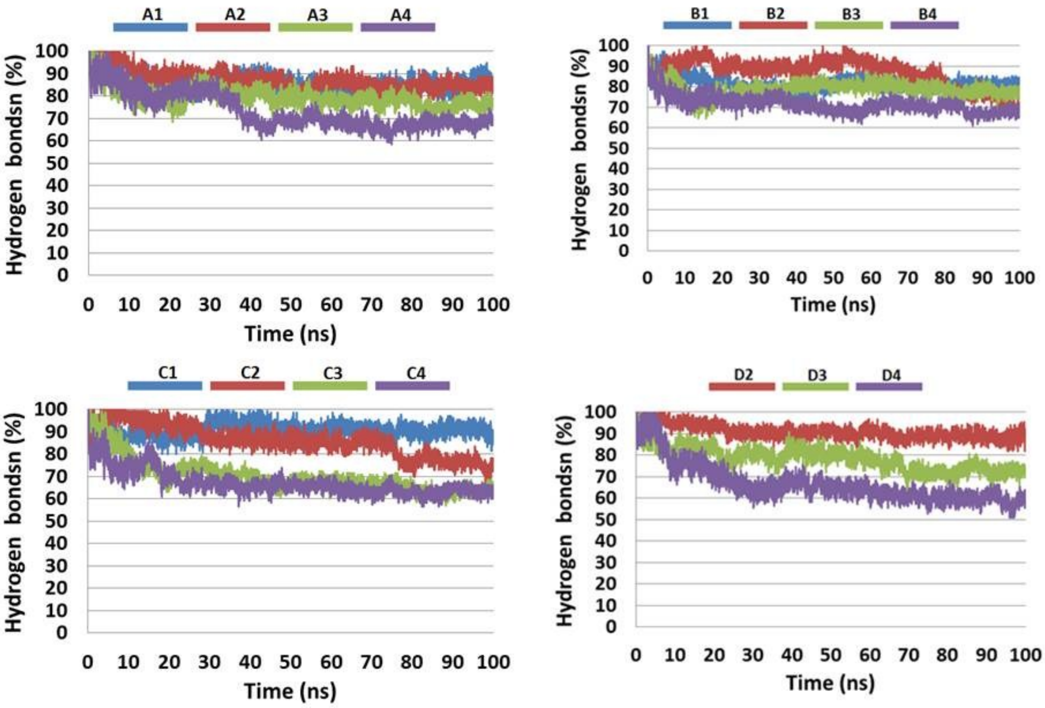

Figure S19: The fraction of the number of hydrogen bonds (in percentage) between all β-strands in amylin aggregates compare to the number in the initial constructed models.

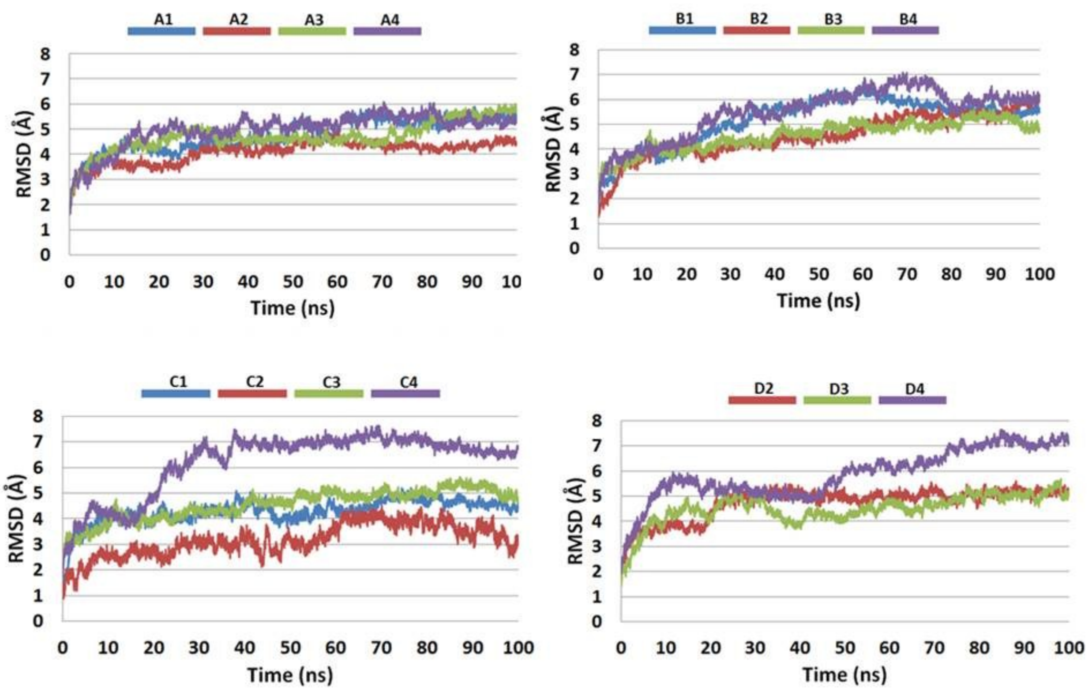

Figure S20: RMSDs of amylin aggregates in insulin-amylin aggregate.

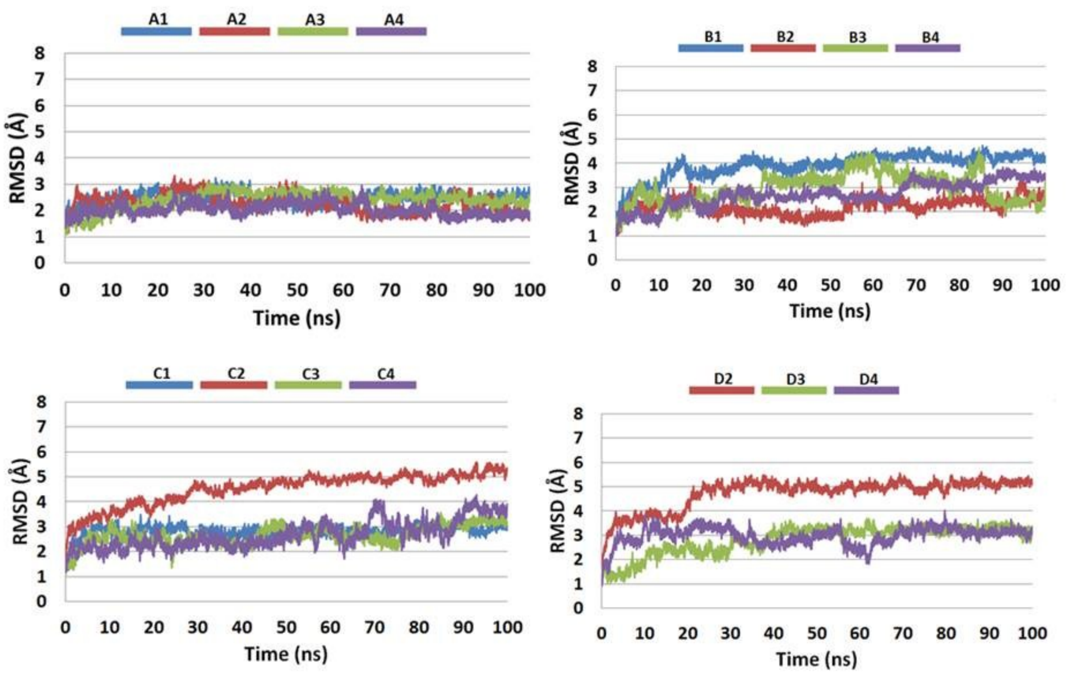

Figure 21: RMSDs of insulin in insulin-amylin aggregates.

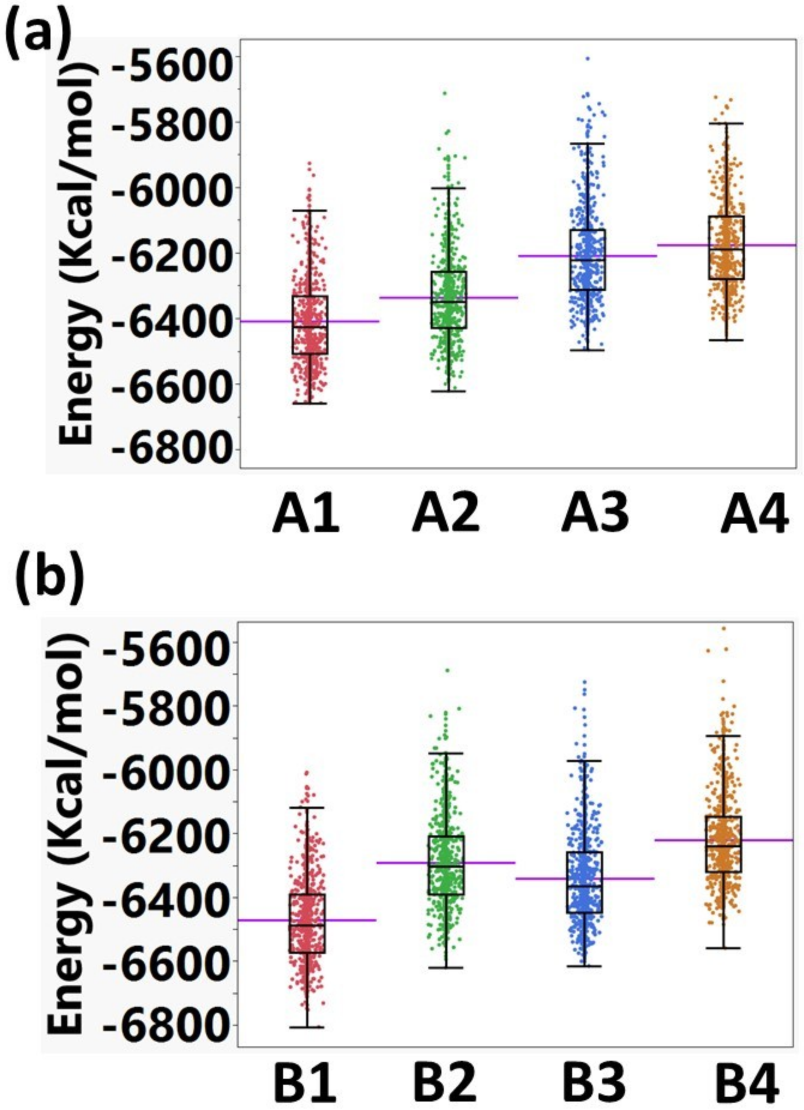

Figure S22: Scatter charts of the 1000 conformations obtained from the GBMV energy values extracted from the last 10 ns of each model (a) A1-A4, and (c) B1 and B4. The scatter charts represent the "histograms" of the number of conformations in energies' range. The averaged energy values are seen in the "boxes". The P values for models A1-A4: $P_{A1,A2}$= 7.35x10^{-28}, $P_{A2,A3}$= 5.99x10^{-66}, $P_{A3,A4}$= 7.76 x10^{-6}; The P values for models B1-B4: $P_{B1,B2}$= 1.55x10^{-111}, $P_{A2,A3}$= 1.70x10^{-14}, $P_{A3,A4}$= 7.65x10^{-63}.

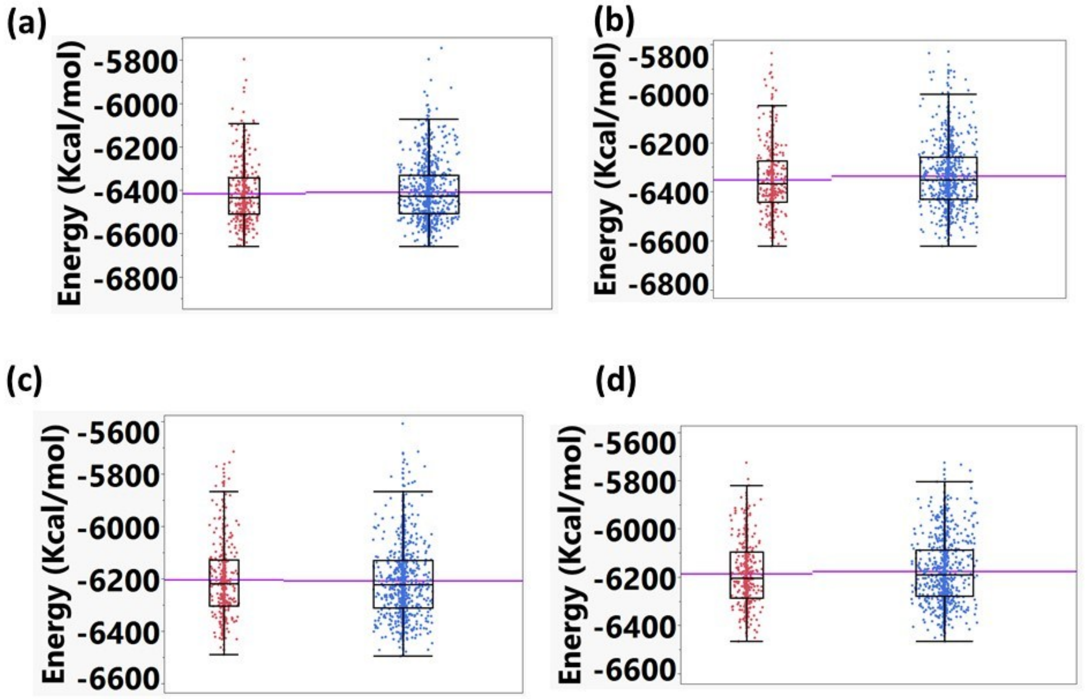

Figure S23: Scatter charts of the 500 conformations (red) versus 1000 conformations (blue) obtained from the GBMV energy values extracted from the last 10 ns of model (a) A1 (b) A2 (c) A3 and (d) A4. The P values between the two statistical calculations for models A1-A4: P_{A1}= 0.99, P_{A2}= 0.099, P_{A3}= 0.92 and P_{A4}= 0.68.

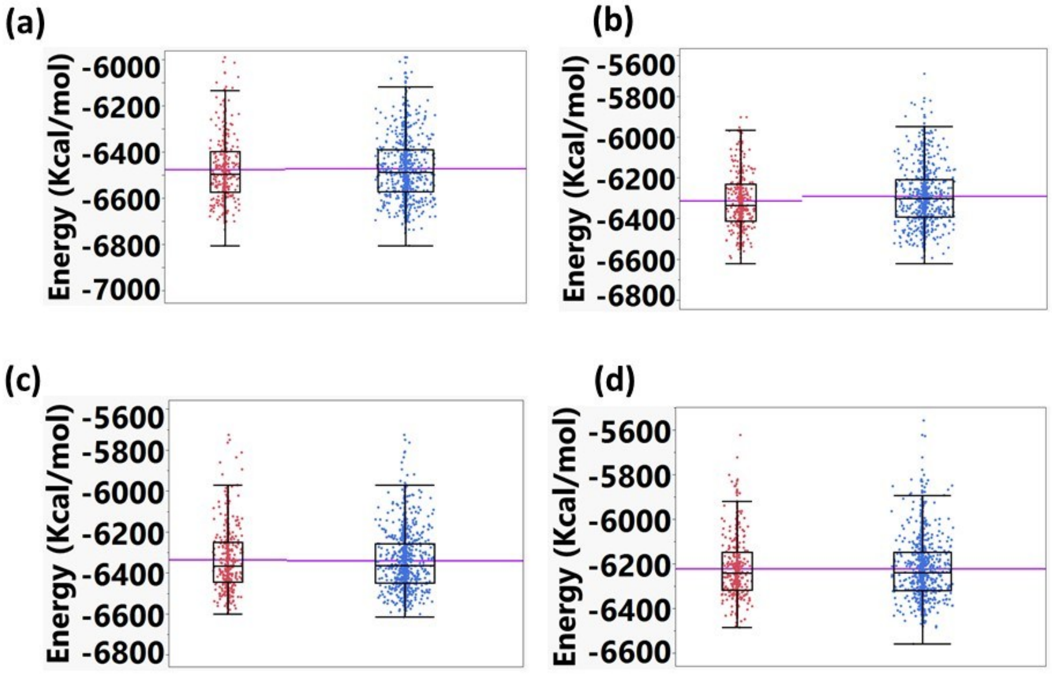

Figure S24: Scatter charts of the 500 conformations (red) versus 1000 conformations (blue) obtained from the GBMV energy values extracted from the last 10 ns of model (a) B1 (b) B2 (c) B3 and (d) B4. The P values between the two statistical calculations for models B1-B4: P_{B1}= 0.71, P_{B2}= 0.014, P_{B3}= 1.00 and P_{B4}= 0.97.

Table S1: The C atoms in the aromatic residues in which the distances between them were measured in order to estimate the π-π interactions between amylin aggregates and insulin B chain (IBC) for wild-type insulin-amylin aggregate models A1, B1 and for models P1 and Q1, in which the Tyr16 in IBC was mutated in Ala.

Model	Residue in amylin	Atom in amylin	Residue in IBC	Atom in IBC
A1	P3(Phe15)	CD1	Tyr16	CE2
B1	P5(Phe15)	CE2	Tyr16	CE2
	P6(Phe15)	CD1	Tyr16	CD2
P1	P3(Phe15)	CZ	Phe24	CE2
	P4(Phe15)	CE2	Phe24	CE1
	P1(Phe15)	CZ	Phe25	CZ
	P2(Phe15)	CD2	Phe25	CZ
	P3(Phe15)	CD2	Phe25	CD2
Q1	P2(Phe15)	CZ	Phe1	CE2
	P3(Phe15)	CZ	Phe1	CE1

Table S2: Description of all simulated models.

Model	System size	Simulation time (ns)
M1	Hexamer	100
M2	Hexamer	100
M3	Hexamer	100
M4	Hexamer	100
Insulin	1 molecule	100
A1	1 insulin molecule and amylin hexamer	100
A2	1 insulin molecule and amylin hexamer	100
A3	1 insulin molecule and amylin hexamer	100
A4	1 insulin molecule and amylin hexamer	100
B1	1 insulin molecule and amylin hexamer	100
B2	1 insulin molecule and amylin hexamer	100
B3	1 insulin molecule and amylin hexamer	100
B4	1 insulin molecule and amylin hexamer	100
C1	1 insulin molecule and amylin hexamer	100
C2	1 insulin molecule and amylin hexamer	100
C3	1 insulin molecule and amylin hexamer	100
C4	1 insulin molecule and amylin hexamer	100
D1	1 insulin molecule and amylin hexamer	100
D2	1 insulin molecule and amylin hexamer	100
D3	1 insulin molecule and amylin hexamer	100
D4	1 insulin molecule and amylin hexamer	100

References:

1. Wineman-Fisher, V.; Atsmon-Raz, Y.; Miller, Y., Orientations of residues along the beta-arch of self-assembled amylin fibril-like structures lead to polymorphism. *Biomacromolecules* 2015, 16, 156-65.
2. Luca, S.; Yau, W. M.; Leapman, R.; Tycko, R., Peptide conformation and supramolecular organization in amylin fibrils: constraints from solid-state NMR. *Biochemistry* 2007, 46, 13505-22.
3. Wiltzius, J. J.; Sievers, S. A.; Sawaya, M. R.; Cascio, D.; Popov, D.; Riekel, C.; Eisenberg, D., Atomic structure of the cross-beta spine of islet amyloid polypeptide (amylin). *Protein science : a publication of the Protein Society* 2008, 17, 1467-74.
4. Brender, J. R.; Krishnamoorthy, J.; Sciacca, M. F.; Vivekanandan, S.; D'Urso, L.; Chen, J.; La Rosa, C.; Ramamoorthy, A., Probing the sources of the apparent irreproducibility of amyloid formation: drastic changes in kinetics and a switch in mechanism due to micellelike oligomer formation at critical concentrations of IAPP. *The journal of physical chemistry. B* 2015, 119, 2886-96.
5. Whittingham, J. L.; Scott, D. J.; Chance, K.; Wilson, A.; Finch, J.; Brange, J.; Guy Dodson, G., Insulin at pH 2: structural analysis of the conditions promoting insulin fibre formation. *Journal of molecular biology* 2002, 318, 479-90.
6. Gilead, S.; Wolfenson, H.; Gazit, E., Molecular mapping of the recognition interface between the islet amyloid polypeptide and insulin. *Angewandte Chemie* 2006, 45, 6476-80.
7. Susa, A. C.; Wu, C.; Bernstein, S. L.; Dupuis, N. F.; Wang, H.; Raleigh, D. P.; Shea, J. E.; Bowers, M. T., Defining the molecular basis of amyloid inhibitors: human islet amyloid polypeptide-insulin interactions. *Journal of the American Chemical Society* 2014, 136, 12912-9.
8. Lee, M. S.; Feig, M.; Salsbury, F. R.; Brooks, C. L., New analytic approximation to the standard molecular volume definition and its application to generalized born calculations. *J Comput Chem* 2003, 24, 1348-1356.
9. Lee, M. S.; Salsbury, F. R.; Brooks, C. L., Novel generalized Born methods. *J Chem Phys* 2002, 116, 10606-10614.
10. Israelachvili, J.; Pashley, R., The hydrophobic interaction is long range, decaying exponentially with distance. *Nature* 1982, 300, 341-2.
11. Kumar, S.; Nussinov, R., Close-range electrostatic interactions in proteins. *Chembiochem : a European journal of chemical biology* 2002, 3, 604-17.
12. Burley, S. K.; Petsko, G. A., Aromatic-aromatic interaction: a mechanism of protein structure stabilization. *Science* 1985, 229, 23-8.
13. Kurochkin, I. V., Insulin-degrading enzyme: embarking on amyloid destruction. *Trends in biochemical sciences* 2001, 26, 421-5.
